# kCSD-python, reliable current source density estimation with quality control

**DOI:** 10.1371/journal.pcbi.1011941

**Published:** 2024-03-14

**Authors:** Chaitanya Chintaluri, Marta Bejtka, Władysław Średniawa, Michał Czerwiński, Jakub M. Dzik, Joanna Jędrzejewska-Szmek, Daniel K. Wójcik

**Affiliations:** Laboratory of Neuroinformatics, Nencki Institute of Experimental Biology of Polish Academy of Sciences, Warsaw, Poland; Ghent University, BELGIUM

## Abstract

Interpretation of extracellular recordings can be challenging due to the long range of electric field. This challenge can be mitigated by estimating the current source density (CSD). Here we introduce kCSD-python, an open Python package implementing Kernel Current Source Density (kCSD) method and related tools to facilitate CSD analysis of experimental data and the interpretation of results. We show how to counter the limitations imposed by noise and assumptions in the method itself. kCSD-python allows CSD estimation for an arbitrary distribution of electrodes in 1D, 2D, and 3D, assuming distributions of sources in tissue, a slice, or in a single cell, and includes a range of diagnostic aids. We demonstrate its features in a Jupyter Notebook tutorial which illustrates a typical analytical workflow and main functionalities useful in validating analysis results.

## Author summary

Recording electric potential with wires inserted in the brain is a standard method in neuroscientist’s arsenal for a century. This extracellular potential reflects electric activity of billions of neural cells processing incoming information and our deepest thoughts. It is difficult to interpret because the effect of every single cell activity propagates in space like a wave in a lake after a stone is thrown. Therefore, every electric recording is a sum of billions of stronger and weaker contributions from close and remote cells, respectively. It is easier to understand the local activity of the brain when we reconstruct the distribution of the sources contributing to the measurements. This is similar to reconstructing the information on where and when stones where thrown into the lake from wiggling of multiple buoys placed on the water surface. The techniques for source reconstruction are called the Current Source Density methods. Early techniques required distributions of electrodes on regular grids. Our kernel Current Source Density method can reconstruct this activity from arbitrary distribution of electrodes and can take into account measurement noise. Here we present our software toolbox, kCSD-python, which implements this method. It comes with a number of tools for efficient estimation, for testing reliability of the results, and with several tutorials showing how to use it.

This is a *PLOS Computational Biology* Software paper.

## Introduction

Extracellular potential recordings are a mainstay of neurophysiology. However, the long range of electric field still makes their interpretation challenging despite decades of research. Extracellular potential in tissue is produced by transmembrane currents. Its low-frequency component, called the Local Field Potential (LFP), is believed to mainly reflect the dendritic processing of synaptic inputs [1, 2]. To facilitate understanding of the processes underlying the recorded signal it is useful to estimate the density of transmembrane current sources (Current Source Density, CSD) [3–8]. CSD gives direct access to physiologically relevant information, which is often concealed in original data [5]. The relation between the CSD and the extracellular potential can be described by the Poisson equation

$$\begin{array}{c} C=-\nabla (\sigma \nabla V), \end{array}$$ (1)

where *C* is the CSD, *V* is the extracellular potential, ∇ is the gradient, and *σ* is the conductivity tensor. For isotropic and homogeneous tissue Eq (1) reduces to

$$\begin{array}{c} C=-\sigma \Delta V \end{array}$$ (2)

where Δ = ∇^2^ is the Laplacian, which can be solved:

$$\begin{array}{c} V\left(x\right)=\frac{1}{4\pi \sigma }\int dx^{\prime }\frac{C(x^{\prime })}{|x-x^{\prime }|}, \end{array}$$ (3)

where $x,x^{\prime }\in R^{3}$. Eqs (2) and (3) show that knowing the potential in the whole space, we can compute the CSD, and knowing the CSD in the whole space, we can compute the potential. Experimentally, we can only access the potential at discrete electrode locations, so direct determination of the CSD in the whole space from Eq (2) is impossible.

To deal with this problem different methods for estimation of current sources have been proposed since the middle of the 20th century [3, 4, 9–14]. Pitts et al. [3] first observed that relation (1) could be used to estimate the sources from measured potential. To investigate the activity in the spinal cord after dorsal root stimulation they proposed to use the second numerical derivative of laplacian for estimation of CSD and made use of approximate translational invariance of potentials along the spinal cord. This approach was further developed in the early 1970s by Freeman, Nicholson, Haberly, and others, which established CSD estimation as a practical approach to investigating relations between field potentials and local neural activity [5].

A significant departure from the previous tradition was the work of Pettersen et al. [9], who proposed a model-based inverse CSD method (iCSD) in 1D. They assumed parametric models of source distribution, e.g. spline interpolated between regularly spaced electrodes, with parameters being the measured potentials, including physical models of source behavior in the dimensions not probed, for example, assuming constant sources within cortical columns. This work was further generalized to 2D and 3D recordings by Łęski et al. [10, 11] but major limitations remained. Those were the requirements of using a regular grid of recordings for estimation and tying estimation space to the experimental setup. Although some approaches to handle simple challenges to irregularity, such as broken contacts or ill recordings, were considered [15], it was only the kernel CSD method [12], generalizing the inverse CSD, which first separated conceptually and computationally the estimation space from the electrode setup. This conceptual separation enabled, for instance, estimation of contributions to LFP from single cells of known morphology [16].

Other model-based approaches were proposed to accommodate specific experimental contexts, such as propagating neuronal activity in the cortex [17, 18], or considered other estimation schemes, e.g. based on Gaussian processes [14]. Kropf and Shmuel [19] investigated properties of inverse and kernel CSD methods from the general perspective of discrete inverse methods and proposed several new variants of these methods. This later inspired the analysis and construction of eigensources, perfectly recoverable sources spanning reconstruction space in kCSD [13].

Despite these conceptual developments not much software is available implementing these different source reconstruction methods. The simplicity of the traditional approach arguably might not require a dedicated package but inclusion of different smoothing approaches of [4] is not available, for example. For the recent model-based methods the inverse CSD papers [9–11] and the kernel CSD [12] were accompanied by MATLAB software which could be used on other data sets. Perhaps a better approach is to use Elephant [Elephant (doi:10.5281/zenodo.1186602; RRID:SCR_003833), Denker 2018], general purpose Python library for analysis of electrophysiological data, which implements inverse CSD, kCSD, and MoIkCSD, as well as many other useful routines. In particular, the kCSD and MoIkCSD implementations in Elephant are derived from early versions of the package we present here. An interesting alternative to kCSD-python could be the GPCSD (Gaussian process current source density estimation) [14]. While conceptually different, the results are effectively consistent with kCSD in the cases tested, although the available functionality of the two packages is different.

**Fig 1:**
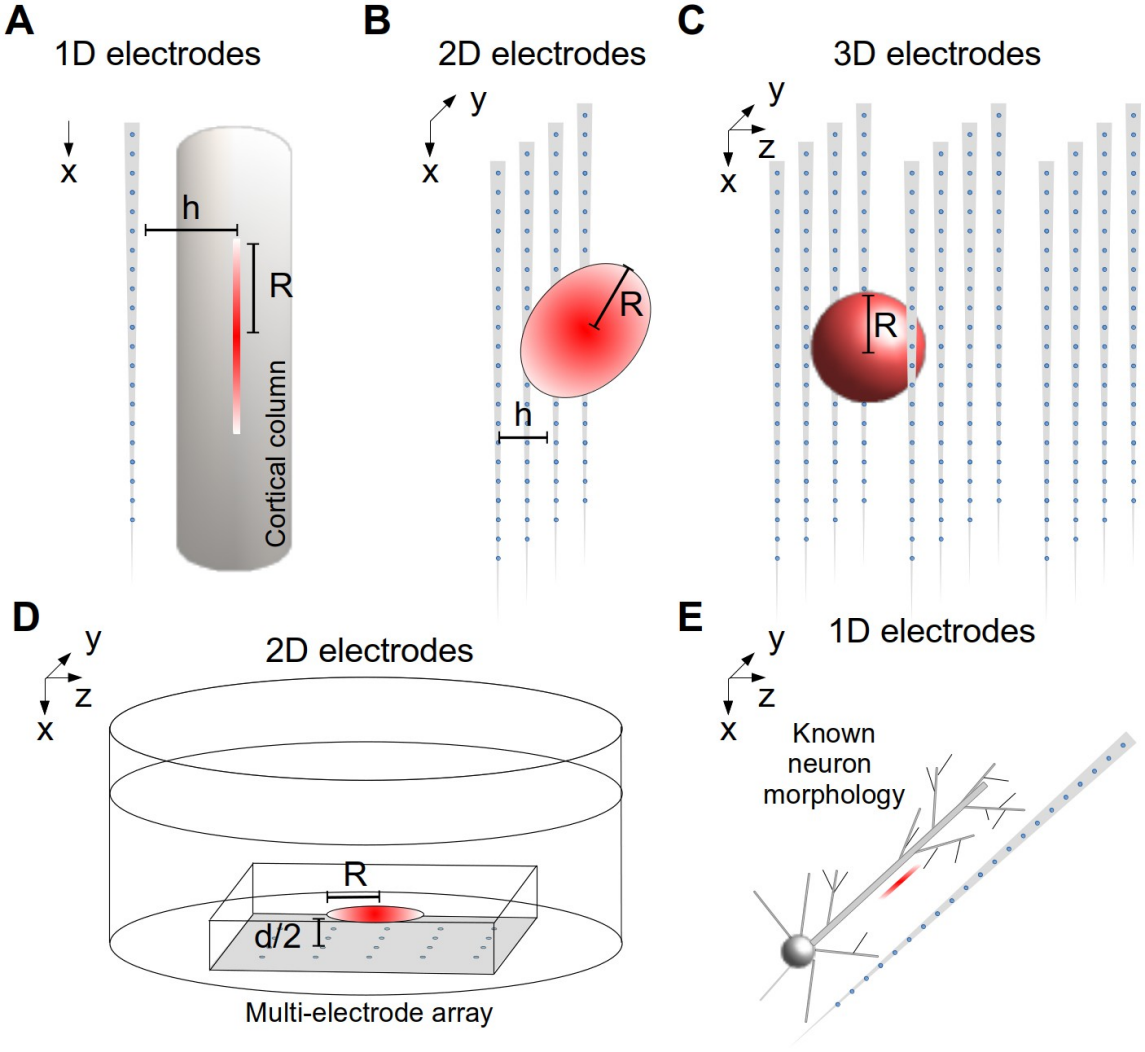
Overview of experimental contexts where kCSD-python is applicable. 1D setups such as A) laminar probes and equivalent (*R*, *h*—radii of the basis source along the electrode and perpendicular to the shaft); 2D setups, such as B) multi-shaft silicon probes, Neuropixel or SiNAPS probes, or D) planar MEA (*R*—radius of the basis source in the plane of the MEA, 2*h*—assumed thickness of the tissue contributing to the measurement, *d*—slice thickness); 3D electrode setups, such as multiple multi-shaft silicon probes, Utah arrays, multiple electrodes placed independently in space with controlled positions (*R*—radius of the basis source), where the sources are assumed to come C) from tissue (kCSD) or E) from single cells with known morphology (skCSD).

Here, we present the first official release of kCSD-python, an open Python toolbox implementing the kernel Current Source Density (kCSD) method [12, 13] and variants [16, 20]. It allows kCSD reconstruction of current sources for data from 1D setups (laminar probes and equivalent electrode distributions), 2D (planar MEA, multi-shaft silicon probes, Neuropixel or SiNAPS probes, etc), and 3D electrode setups (Utah arrays, multiple electrodes placed independently in space with controlled positions), where the sources are assumed to come from tissue (kCSD) or from single cells with known morphology (skCSD, [16]). Fig 1 shows the different experimental scenarios for which this software can be used.

We also include some old and introduce some new diagnostic features which we found useful in CSD analysis and illustrate the application of the package and the new diagnostic tools in typical analysis workflow implemented as a Jupyter notebook [21] tutorial.

For reader convenience we first briefly restate the kCSD method [12, 13]. Then in the Results section we introduce several new diagnostic tools included in the presented package. First, we added the L-curve method for parameter selection. Then, to get informed on reconstruction accuracy we introduce *measurement uncertainty maps*, which show how measurement noise affects the estimated CSD, and *reliability maps*, which help build intuition on the reliability of estimates for classes of possible sources for a given setup. We then briefly introduce the new package which is extensively illustrated in the provided tutorial (S1 Text). This Jupyter Notebook [21] tutorial, which is part of the toolbox, is provided to facilitate understanding and usage of the kCSD method. The tutorial enables the user to analyze the CSD using simulated surrogate data (electrode positions and recordings) or actual recordings.

## Design and implementation

### Overview of kernel Current Source Density method

Here we review basic aspects of the kernel Current Source Density method. For details and proofs see [12, 13]. Assume we have *N* electrodes placed at $x_{j}\in R^{k}$, *j* = 1, …, *N*, *k* ∈ {1, 2, 3}. To estimate CSD in the space of relevant dimension we cover the region of interest with a family of CSD sources which we call basis sources, ${\tilde{b}}_{j}\left(x\right)$; think of many little Gaussians. They come with corresponding basis sources in the potential space, *b*_*j*_(**x**), which are contributions to the potential from ${\tilde{b}}_{j}\left(x\right)$ where specific functional relation between them depends on the dimensionality of space and assumed model of tissue, see below for examples. These basis functions span a family of CSD and corresponding potential functions which can be expressed as

$$\begin{array}{ccc} C(x) & = & \alpha_{1}{\tilde{b}}_{1}\left(x\right)+\cdots +\alpha_{M}{\tilde{b}}_{M}\left(x\right), \end{array}$$ (4)

$$\begin{array}{ccc} V(x) & = & \alpha_{1}b_{1}\left(x\right)+\cdots +\alpha_{M}b_{M}\left(x\right). \end{array}$$ (5)

Using the basis sources we construct two functions which we call kernel and cross-kernel functions [12, 13]

$$\begin{array}{c} K\left(x,x^{\prime }\right)=\sum_{i=1}^{M}b_{i}\left(x\right)b_{i}\left(x^{\prime }\right),\;\;\tilde{K}\left(x,y\right)=\sum_{i=1}^{M}{\tilde{b}}_{i}\left(x\right)b_{i}\left(y\right). \end{array}$$ (6)

Clearly, *K* is symmetric while $\tilde{K}$ is not. With these kernel functions any CSD and corresponding potential distributions can be written as

$$\begin{array}{ccc} V(x) & = & \sum_{j=1}^{L}\beta_{j}K\left(x,x_{j}\right) \end{array}$$ (7)

$$\begin{array}{ccc} C(x) & = & \sum_{j=1}^{L}\beta_{j}\tilde{K}\left(x,x_{j}\right) \end{array}$$ (8)

for some *L*, *β*_*j*_, **x**_*j*_ [12]. The first step of kernel Current Source Density method is kernel interpolation of the measured potential with kernel *K*. To avoid overfitting, correction for noise is made by minimizing prediction error

$$\begin{array}{c} \text{err}\left[V\right]=\sum_{i=1}^{N}{\left(V^{*}\left(x_{i}\right)-V_{i}\right)}^{2}+\lambda {\left|\beta \right|}^{2}. \end{array}$$ (9)

From the Representer Theorem [22] we know this is minimized by Eq 7 with *L* = *N* and **x**_*j*_ the electrode positions

$$\begin{array}{c} V^{*}\left(x\right)=\sum_{j=1}^{N}\beta_{j}K\left(x,x_{j}\right), \end{array}$$ (10)

which gives

$$\begin{array}{c} \beta ={\left(K+\lambda I\right)}^{-1}V, \end{array}$$ (11)

where **V** is the vector of measured potentials *V*_*i*_, λ is regularization parameter, and

$$\begin{array}{c} K_{i,j}\equiv K\left(x_{i},x_{j}\right). \end{array}$$ (12)

In the second step, we use cross-kernel $\tilde{K}\left(x,x^{\prime }\right)$, to estimate the CSD:

$$\begin{array}{c} C^{*}=\tilde{K}{\left(K+\lambda I\right)}^{-1}V. \end{array}$$ (13)

We use * to indicate estimated models of potential and sources.

We select the basis source functions ${\tilde{b}}_{i}$ so that they are convenient to work with numerically. Usually they are step functions or Gaussians with non-trivial support over regions which are most natural for the problem at hand. Specific functional relation between *b*_*i*_ and ${\tilde{b}}_{i}$. depends on the problem at hand, the dimensionality of space in which estimation is desired, as well as on physical models of the medium, such as tissue conductivity, slice or brain geometry, etc. [9–11, 16, 20]. An overview relevant to the implementation of kCSD-python is provided in the next section.

We considered CSD reconstruction for recordings from 1D (Fig 1A), 2D (Fig 1B) and 3D setups (Fig 1C) under assumption of infinite tissue of constant conductivity [12], we used method of images to improve reconstruction for slices of finite thickness on MEA under medium of different conductivity (ACSF, [20]), (Fig 1D), and we considered reconstruction of sources along single cells when we have reasons to trust the recorded signal to come from a specific cell of known morphology [16], (Fig 1E). All these variants are implemented in the present code. Fig 1 shows these scenarios.

In practical applications the challenges of the method include selection of basis and the relevant parameters as well as reliability of the estimation. Some techniques were discussed before [12, 13]. Here we introduce more techniques for parameter selection and for evaluating reconstruction reliability, and we illustrate both new and old approaches with the provided package.

### Basis sources in the source and potential spaces

In the simplest case of infinite, homogeneous and isotropic tissue in 3D (Fig 1C) we have

$$\begin{array}{c} b_{i}\left(x,y,z\right)=\frac{1}{4\pi \sigma }\int dx^{\prime }\int dy^{\prime }\int dz^{\prime }\;\frac{{\tilde{b}}_{i}\left(x^{\prime },y^{\prime },z^{\prime }\right)}{\sqrt{{\left(x-x^{\prime }\right)}^{2}+{\left(y-y^{\prime }\right)}^{2}+{\left(z-z^{\prime }\right)}^{2}}}. \end{array}$$ (14)

In general, we can consider arbitrary conductivity and geometry of the tissue which may force us to use approximate numerical methods, such as finite element schemes. For example, [20] show an application of kCSD for a slice of finite thickness and specific geometry, as well as a method of images approximation for kCSD for typical slices on multielectrode arrays (recordings far from the boundary, slice much thinner than its planar extent), Fig 1D.

For laminar probes, Fig 1A), following [9], we assumed elementary current sources contributing to the potential of the form ${\tilde{b}}_{i}\left(z\right)H\left(x,y\right)$. Here ${\tilde{b}}_{i}\left(z\right)$ is the one-dimensional basis source (we usually assume a Gaussian of radius *R*). Since information beyond the electrode axis is unavailable we assume rotational symmetry around *z*. We usually assume *H*(*x*, *y*) a step function on a disk of radius *h*:

$$\begin{array}{c} H\left(x,y\right)=\{\begin{array}{cc} 1 & x^{2}+y^{2}⩽h^{2},\\ 0 & \text{otherwise}. \end{array} \end{array}$$

This can be integrated yielding 1D potential basis functions of the form

$$\begin{array}{c} b_{i}\left(z\right)=\frac{1}{2\sigma }\int dz^{\prime }\;(\sqrt{{\left(z-z^{\prime }\right)}^{2}+h^{2}}-|z-z^{\prime }|){\tilde{b}}_{i}\left(z^{\prime }\right). \end{array}$$ (15)

For planar setups, Fig 1B) [11], we usually assume Gaussian basis sources ${\tilde{b}}_{i}\left(x,y\right)$, physically contributing to the potential with ${\tilde{b}}_{i}\left(x,y\right)H\left(z\right)$, where

$$\begin{array}{c} H\left(z\right)=\{\begin{array}{cc} 1 & -h⩽z⩽h\\ 0 & \text{otherwise}. \end{array} \end{array}$$

This can be integrated to give the potential in the electrode plane:

$$\begin{array}{c} b_{i}\left(x,y\right)=\frac{1}{2\pi \sigma }\int dx^{\prime }\int dy^{\prime }\;\text{arsinh}(\frac{2h}{\sqrt{{\left(x-x^{\prime }\right)}^{2}+{\left(y-y^{\prime }\right)}^{2}}}){\tilde{b}}_{i}\left(x^{\prime },y^{\prime }\right). \end{array}$$ (16)

This approach gives two parameters describing the CSD basis functions, the radius of the relevant Gaussian, *R*, and the thickness of contributing layer in 2D case or radius of circular sheath in 1D case (*h*). Note that if we assume above *H*(*x*, *y*) and *H*(*z*) to be Gaussian as well with the same radius, in all three dimensionalities the individual contributions are spherically symmetrical Gaussians. Therefore, the same 3D approach can be used. Further, it can be integrated to yield potential in a closed form. Indeed, from Eq (14), taking

$$\begin{array}{c} {\tilde{b}}_{j}\left(\bar{x}\right)=\frac{1}{{\left(\sqrt{2\pi }R\right)}^{3}}\text{exp}\frac{-{\left(\bar{x}-\bar{x_{j}}\right)}^{2}}{2R^{2}} \end{array}$$

we can show that

$$\begin{array}{c} b_{j}\left(\bar{x}\right)=\frac{1}{4\pi \sigma |\bar{x}-{\bar{x}}_{j}|}\text{erf}(\frac{|\bar{x}-{\bar{x}}_{j}|}{\sqrt{2}R}) \end{array}$$

where

$$\begin{array}{c} \text{erf}\left(r\right)=\frac{2}{\sqrt[{}]{\pi }}\int_{0}^{r}e^{-t^{2}}dt. \end{array}$$

This is also implemented in the present code.

### Overview of the implementation

Here we focus on the code organization in the kCSD-python package which is a Python implementation of the kernel Current Source Density method [12] and its two variants ([20] and [16]). Mathematical details of the kCSD algorithm are described above. The recommended workflow and practical usage of the majority of tools from kCSD-python is presented in the supplementary tutorial (S1 Text). Additional online tutorials and scripts generating all the figures from this article provide more help; see section on code availability below.

The package is divided into four main modules:

- KCSD—the core of the package. It is used to generate Current Source Density estimates using kCSD method for a given configuration of electrode positions and recorded potentials (in practice for 1D, 2D and 3D setups, as described in [12, 20]). Useful for the analysis of experimental data.
- sKCSD—generates Current Source Density estimates for a single neuron with known morphology using skCSD method [16]; Exemplary usage is shown in an online tutorial available at https://kcsd-python.readthedocs.io/en/latest/TUTORIALS.html#skcsd-tutorial.
- ValidateKCSD—implements classes useful for validation of the quality of kCSD method estimates. It is used for generation and analysis of surrogate data.
- VisibilityMap—implements classes generating reliability maps for a given configuration of electrode positions. Exemplary usage is shown in an online tutorial available at https://kcsd-python.readthedocs.io/en/latest/TUTORIALS.html#advanced-features.

Within the KCSD module there is a base class for all kCSD variants called KCSD. Depending on the different physical assumptions and the dimensionality of electrode distribution, which affects the relations between the basis sources in the potential and CSD space (see S1 Text), the following derived classes are provided:

- KCSD1D, KCSD2D, KCSD3D [12]—estimate the Current Source Density, for a given configuration of electrode positions and recorded potentials, in the case of 1D, 2D and 3D recording electrodes, respectively. Using these classes the region of interest, which is spanned by the electrode positions by default, is covered by regularly placed basis sources ${\tilde{b}}_{j}\left(x\right)$. Estimation points, locations in which reconstructed CSD is obtained, lie on a regular grid too. Inherit KCSD class.
- MoIKCSD—This estimates the Current Source Density, for a given configuration of electrode positions and recorded potentials, in the case of 2D recording electrodes from a MEA electrode plane using the Method of Images [20]. Inherits KCSD2D class.
- oKCSD1D, oKCSD2D, oKCSD3D—estimate the CSD in explicitly specified region. Classes require to specify particular locations (not necessarily regular) where to place basis sources ${\tilde{b}}_{j}\left(x\right)$ and also where to estimate the solution. Inherit KCSD1D, KCSD2D and KCSD3D class respectively.

For all those classes the workflow is similar. To reconstruct the CSD for a given electrode setup the user needs to call an appropriate class providing the electrode positions and the recorded potentials as arguments. One may provide additional parameters determining basis sources definition and placement (number, placement, shape/type) and which specify the estimation space. During the class call the key objects such as the kernel matrix *K* (Eq 6) and the cross-kernel matrix are created. Based on them the estimated CSD is obtained. S1 Text illustrates example usage of the kCSD-python package using simple 2D electrode setup and KCSD2D class.

## Results

### Source reconstruction with kCSD from experimental data

Kernel CSD, just like other CSD estimation methods, is a tool helping interpret field potential recordings. Its main advantages over previous methods are self-consistent CSD distributions in whole brain regions, ease of estimation from arbitrarily distributed contacts, possibility of restricting sources to specific regions, and separation of experimental setup (electrode distribution) from analytical setup (choice and distribution of basis sources). It was used in several projects where this flexibility was advantageous [13, 14, 16, 23–31].

A common usage of CSD analysis is identification of border layers in laminar structures, and kCSD was also applied to that purpose, in particular in the studies of cortical activity and the structure of the olfactory bulb. Kernel CSD was used extensively by Bijanzadeh, Nurminen et al. [24, 25] in their study of distinct laminar processing of local and global context in primate primary visual cortex. They computed CSD from the band-pass filtered (1–100Hz) and trial-averaged LFPs. They used CSD responses to small stimuli flashed inside the receptive fields to identify laminar borders, localize surround-evoked input activity to specific cortical layers, study the laminar location of the subthreshold inputs, and measure the onset latency of the surround stimuli. Sederberg et al. [27] used CSD analysis to functionally determine cortical layers of the awake mouse from the average stimulus-evoked response, and to analyze the pre-stimulus activity (in single trials) to localize sinks and sources generating the predictive signal used in their classifiers of awareness states. Their main reason for selecting kCSD was the ease of handling irregular spacing between electrodes occurring even on regular grids of electrodes when certain contacts do not satisfy quality control. Similarly, in their studies of activity in the barrel cortex related to trace eyeblink conditioning, Silva-Prieto et al. [31] used 2D kCSD to facilitate identification of borders of cortical columns and layers.

After identifying the olfactory bulb as a main source of high-frequency oscillations (130–180 Hz) associated with a subanesthetic dose of ketamine in rodents, Hunt et al. [26] used kCSD to localize laminar borders within the bulb, attribute the activity to specific cell types, and to precisely estimate phase shifts between oscillations in OB and ventral striatum. In a systematic study of large-scale spatiotemporal circuit information within the olfactory bulb with a high-density CMOS chip, Hu et al. [30] used kCSD to identify the fine details of sources and sinks activity during the global activation of the entire OB circuit.

Some less standard applications of kCSD focused on the search for dominating sources of specific LFP activity or combined this method with other analytical approaches. Studying GABAergic inhibition effects on interictal dynamics in awake epileptic mice, Muldoon et al. [23] used kCSD to consistently reveal a source in the CA1 pyramidal cell layer. Somewhat surprisingly they found a correlation between the maximum value of the kCSD source located in the pyramidal layer and the amplitude of interictal spikes recorded at the global/surface level from the contralateral EEG. Fedor et al. [28] used kCSD to build a substrate closer to true dynamics to establish a functional coherence map for microECoG recordings in a rat schizophrenia model. Of particular use was the deblurring or deconvolving effect of CSD analysis. From that perspective, it was similar in spirit to the work of Klein et al. [14], who used their own Gaussian Process CSD method (GPCSD), largely consistent with kCSD, to estimate cortical layer-specific phase coupling between two probes and showed that the same analysis applied directly to LFPs did not recover these patterns.

The flexibility of basis source placement and electrode distribution as well as their conceptual and analytical independence inherent to kCSD allowed Cserpan et al. [16]) analysis of the distribution of current sources along a known morphology of a hippocampal neuron from LFPs recorded with a set of metal electrodes triggered on spikes of the studied cell (single cell kCSD; sckCSD).

We recently showed how kCSD can be used in relatively common but non-obvious case of recordings with Neuropixels probe [13]. This is an interesting case because the electrode positions do not span a regular 2D grid as considered in previous methods, such as traditional or inverse CSD (iCSD). Also, the quasi-linear design with checker-board design of effectively 4 nearby linear probes situates this design on the border of 1D and 2D probes. Previously, such a design would stimulate various ad hoc approaches to CSD analyses. With kCSD, which separates conceptually and computationally estimation space from recording electrodes, it is easy and natural to test different hypotheses of what is the effect of different analytical decisions. [13] show that 2D analysis of Neuropixels recordings can in fact provide non-trivial 2D information pointing to possible microcolumns within the barrel cortex while the eigensources analysis allows to compare reliability of information recovered in the different directions, with horizontal direction resolved only with 34th and 36th eigensources [13].

In [29] we used the kCSD method to accurately identify the spatial and temporal profiles of source dynamics in different relevant bands of LFP activity within the rat olfactory bulb. In slow frequencies (0.3–3 Hz) related to breathing we observed dipolar activity propagating from glomerular layer, before emergence of 80–130 Hz activity, to EPL layer, as the 80–130 Hz power rises in time. We found strong dipoles around the mitral layer in the high frequency band.

### Parameter selection

An important part of kCSD estimation is selection of parameters, in particular the regularization parameter, λ, but also the radius of the basis source, *R*. Previously we proposed to use cross-validation [12]. Here we introduce L-curve approach [19, 32] for regularization. Both these methods are implemented in kCSD-Python.

#### Cross-validation

To select parameters using cross-validation [12] we consider a range of parameter values, λ ∈ [λ_0_, λ_1_]. For any test value λ we select an electrode *i* = 1, …, *N* and ignore it. With Eq (10) we build a model from the remaining measurements, $V_{\lambda }^{\prime i}\left(x\right)$, and use it to predict the value at the ignored electrode, $V_{\lambda }^{\prime i}\left(x_{i}\right)$. Here

$$\begin{array}{c} V_{\lambda }^{\prime i}\left(x\right)=\sum_{j\neq i}\beta_{\lambda ,j}^{\prime i}K\left(x,x_{j}\right), \end{array}$$ (17)

where the minimizing vector

$$\begin{array}{c} \beta_{\lambda }^{\prime i}={\left(K^{\prime i}+\lambda I^{\prime i}\right)}^{-1}V_{\lambda }^{\prime i}, \end{array}$$ (18)

and where ′^*i*^ means *i*-th column and row are removed from the given matrix (or vector). We repeat this for all the electrodes *i* = 1, …, *N* and compare predictions from the remaining electrodes against actual measurements:

$$\begin{array}{c} \text{prediction}\;\text{error}\left(\lambda \right)=\sqrt{\sum_{i=1}^{N}{\left(V_{\lambda }^{\prime i}\left(x_{i}\right)-V_{i}\right)}^{2}}. \end{array}$$ (19)

For the final analysis, λ giving minimum prediction error is selected. It is worth checking if the global minimum is also a local minimum. If the λ selected is one of the limiting values this may indicate that extending the range of λ might give a better result or that the problem is ill-conditioned, for example too noisy, and we are either underfitting or overfitting, as we discuss below for the L-curve. As a rule of thumb, the range of tested λ parameters should cover eigenvalues of the (12).

#### L-curve

**Fig 2:**
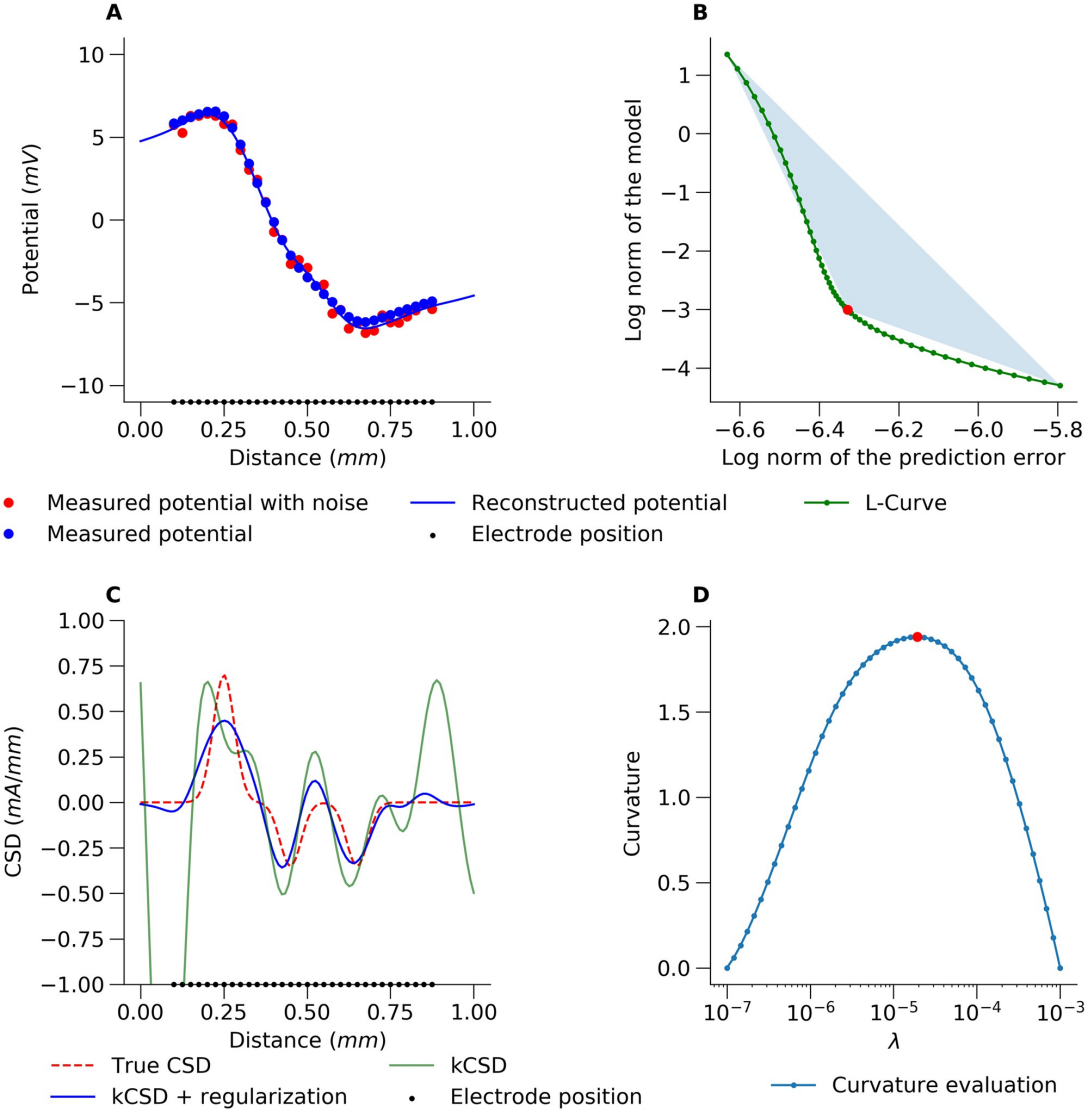
An example of the L-curve method for estimating kCSD parameters. A) The red points represent the potential used for CSD reconstruction. The black points show the electrode positions. The ground truth is shown in panel C with the red dashed curve. The measurement was simulated by adding small random noise to all the electrodes (32 values taken from a uniform distribution). The blue line shows a kernel interpolation of the potential which is the first step of kCSD method. B) L-curve plot for a single R parameter. The apex of the L-curve is numerically computed from the oriented area of directed triangles connecting the point on the L-curve with its two ends. C) Comparison of the true CSD and kCSD reconstruction for parameters obtained with L-curve regularization. D) Estimation of L-curve curvature with triangle method (see the Methods).

Consider the error function, Eq (9), which we minimize to get the regularized solution, *V*_λ_ = **K** *β*_λ_ It is a sum of two terms we are simultaneously minimizing, prediction error

$$\begin{array}{c} \varrho_{\lambda }=\sum_{i=1}^{N}{\left(V_{\lambda }\left(x_{i}\right)-V_{i}\right)}^{2}, \end{array}$$ (20)

and the norm of the model

$$\begin{array}{c} \eta_{\lambda }=∥V_{\lambda }{\left(x\right)∥}_{F}^{2}=\left|\beta_{\lambda }^{T}K\beta_{\lambda }\right|, \end{array}$$ (21)

weighted with λ. Taking λ = 0 is equivalent to assuming noise-free data. In this case we are fitting the model to the data, in practice, overfitting. On the other hand, taking large λ means assuming very noisy data, and in practice ignoring measurements, which results in a flat, underfitted solution. Between these extremes there is usually a solution such that if we decrease λ, the prediction error, ϱ, slightly decreases, while the norm of the model, *η*, increases fast, and if we increase λ, the prediction error, ϱ, increases fast, while the norm of the model, *η*, slightly decreases; see Fig 2D.

This is apparent when the prediction error and the norm of the model are plotted in the log-log scale. This curve follows the shape of the letter L, hence the name L-curve [33]. Several methods have been proposed to measure the curvature of the L-curve and to identify optimal parameters [34]. In kCSD-python, we have implemented the triangle area method proposed by [35]. To distinguish between convex and concave plots, the clockwise directed triangle area is measured as negative Fig 2D shows this estimated curvature for our example as a function of λ.

To illustrate this method in the context of CSD reconstructions, we study an example of 1D dipolar current source with a split negative pole (sink; see Fig 2C, True CSD, red dashed line). We compute the potential at 32 electrodes (Fig 2A and 2C, black dots) with additive noise at every electrode. Notice that if we want to interpret the recorded potential directly (Fig 2A, red dots) it is difficult to discern the split sink. Fig 2D shows the estimated curvature for our example as a function of λ. The optimal value of λ is found by maximizing the curvature of the log-log plot of *η* versus ϱ, Fig 2B. The red dot in Fig 2B and 2D, indicates the ideal λ parameter for this setup obtained through the L-curve method.

#### Selection of multiple parameters

Often we need to tune not just λ but also other parameters. For example, for Gaussian basis sources we may want to decide on the width of the Gaussian used, *R*. To obtain the optimal set of parameters in that case we compute the curvature of the L-curve or the cross-validation error for some ranges of parameters considered and select parameters corresponding to the maximum curvature / minimum error in the parameter space. This is a simplification of the proposition by [36] which in practice we found very effective.

**Fig 3:**
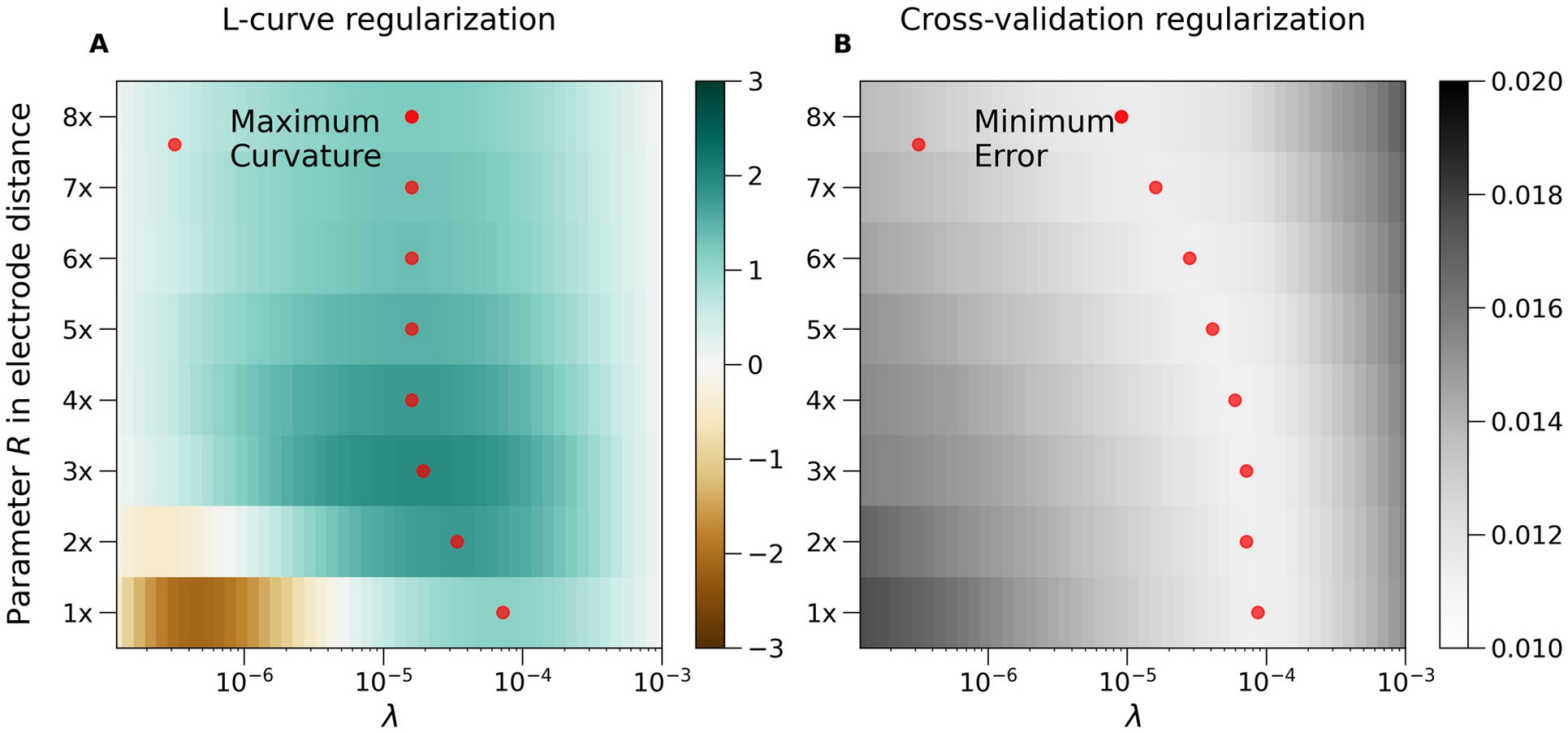
A) L-curve curvature and B) CV-error for the problem studied in Fig 2. Observe that in both cases there are ranges of promising candidate parameter pairs, *R*, λ, which can give good reconstruction given the measured data. Red dots shows local extrema for each value of *R* fixed. See text for discussion of this effect.

As an example, in Fig 3. we show the results of such a scan for the problem shown in Fig 2. The range of λ to be considered can be set by hand but by default we base it on the eigenvalues of *K*. The smallest λ is set as the minimum eigenvalue of *K* which here was around 1e-10. We set maximum λ at the standard deviation of the eigenvalues, which here was around 1e-3. The range of *R* values studied was from the minimum interelectrode distance to half the maximum interelectrode distance. Note that for very inhomogeneous distributions of electrodes this approach may be inadequate.

What we find is that apart from a global minimum in *R*, λ space there is a range of *R* values fixing which we can find optimal λ(*R*) which leads to very close curvatures / CV-errors / estimation results. What happens is that within some limits we may achieve similar smoothing effects changing either λ or *R*. Bigger λ means more smoothing, but bigger *R* means broader basis functions and effectively also smoother reconstruction space. This is why the CV-error and curvature landscapes are relatively flat, or have these marked valleys observed in Fig 3. This effect supports the robustness of the kCSD approach.

### Reconstruction accuracy

With the kCSD procedure one can easily estimate optimal CSD consistent with the obtained data. However, so far we have not discussed the estimation of errors in the reconstruction. Since the errors may be due to several factors—the procedure itself, measurement noise, incorrect assumptions—one may approach this challenge in different ways.

First, to understand the effects of the selected basis sources and setup, one may consider the estimation operator $\tilde{K}{\left(K+\lambda \right)}^{-1}$ and the space of solutions it spans. This space is given by the eigensources, introduced and described thoroughly in [13]. The orthogonal complement of this space in the original estimation space, spanned by ${\tilde{b}}_{j}\left(x\right)$ basis functions is not accessible to the kCSD method. The study of eigensources facilitates understanding which CSD features can be reconstructed and which are inaccessible.

Second, to consider the impact of the measurement noise on the reconstruction, for any specific recording consider the following model-based procedure. Reconstruct CSD from data with optimal parameters. Compute potential from estimated CSD. Add random noise to each computed potential. The noise could be estimated from data, either as a measure of fluctuations on a given electrode for a running signal, or from variability of evoked potentials. Then, for any realization of noise, compute the estimation of CSD. The pool of estimated CSD gives an estimation of the error at any given point where the estimation is made.

**Fig 4:**
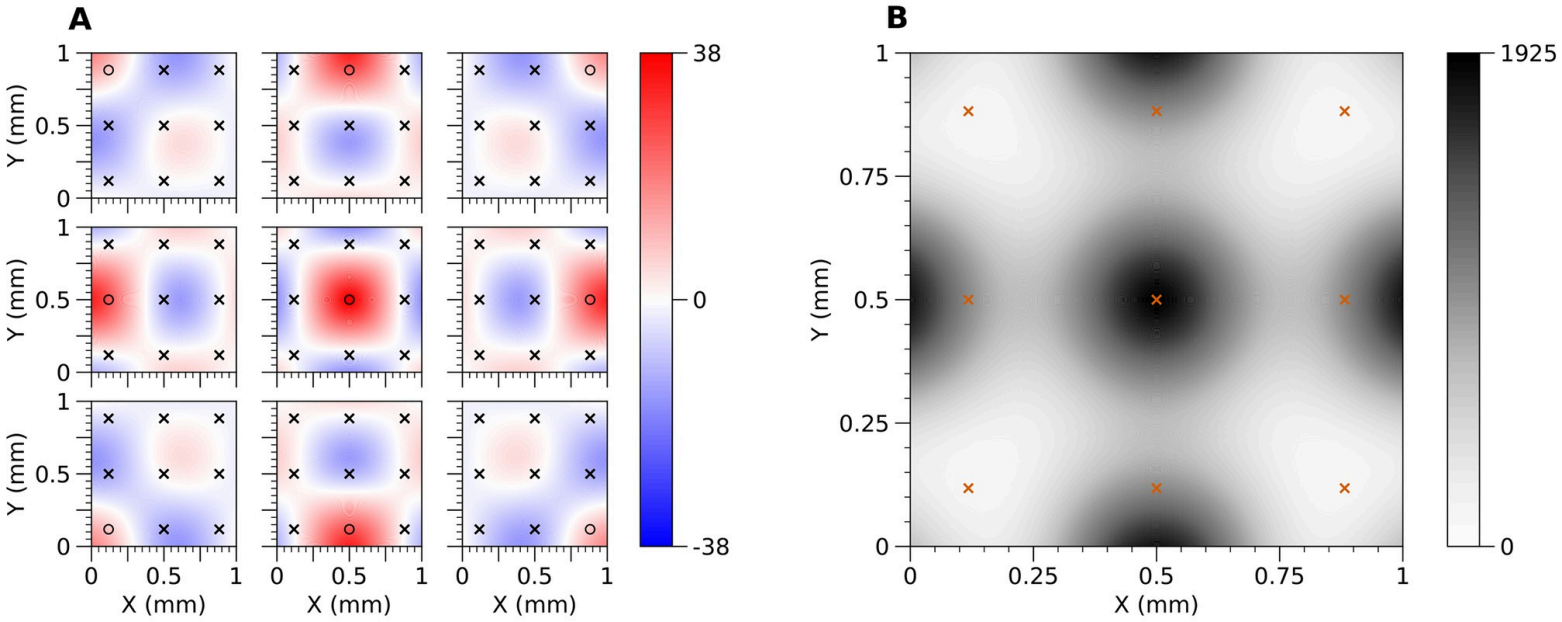
A) Error propagation maps for 3 × 3 regular grid of electrodes. Every panel represents the contribution of the potential measured at the corresponding electrode marked with a black circle (°) to the reconstructed CSD. Every other electrode is marked with a black cross (×). B) Map of CSD measurement uncertainty for 3 × 3 regular grid of electrodes. The CSD measurement uncertainty is represented by variance of the CSD reconstruction caused by the uncertainty in measurement of the potentials. It is assumed that measurement errors for electrodes are mutually independent and follow standard normal distribution ($\varepsilon_{i}∼N\left(0,1\right)$). Location of electrodes is marked with red crosses (×).

This computation can be much simplified by taking advantage of the linearity of the resolvent, $E=\tilde{K}{\left(K+\lambda I\right)}^{-1}$. Then, the *i*-th column (**E**_*i*_) represents contribution of unitary change of *i*-th measured potential (the *i*-th element of the vector **V**) to the estimated CSD (${\underline{C}}^{*}$). As the contribution is proportional to the change, the column can be considered an *Error Propagation Map* for *i*-th measurement (Fig 4A). Note that these vectors (the columns of the resolvent, **E**_*i*_) also happen to form another basis of the solution space, an alternative to the basis of eigensources.

If *ε*_*i*_ is an error of *i*-th measurement, then its contribution to ${\underline{C}}^{*}$ is *ε*_*i*_**E**_*i*_. Moreover, if the measurement errors follow multivariate normal

$$\begin{array}{c} \varepsilon ∼N(0,\Sigma_{V}), \end{array}$$ (22)

then

$$\begin{array}{c} V∼N(V_{exact},\Sigma_{V}), \end{array}$$ (23)

and the estimated CSD also follows multivariate normal

$$\begin{array}{c} {\underline{C}}^{*}∼N\left(EV_{exact},E\Sigma_{V}E^{T}\right). \end{array}$$ (24)

The diagonal of **E** Σ_*V*_**E**^*T*^ represents a *map of CSD measurement uncertainty* (uncertainty attributed to the noise in the measurement, Fig 4B). In the special case when *ε*_*i*_ are mutually independent and of equal variance *σ*^2^, the map of CSD measurement uncertainty can be calculated as a diagonal of $\text{Cov}\left[{\underline{C}}^{*}\right]=EE^{T}\sigma^{2}$.

Third, one can study reconstruction accuracy for a meaningful family of test functions. This could be the Fourier modes for rectangular regions or a collection of Gaussian test functions, centered in different places, of single or multiple radii. For each of these test functions one would compute the potential, perform reconstruction, and compare the results with the original at every point. Finally, one could average this information over multiple different test sources computing a single *Reliability Map*, which we now introduce.

#### Reliability maps

Assume the standard kCSD setup, that is a region $R\subset R^{n}$ where we want to estimate the sources, set of electrode positions, **x**_*i*_, and perhaps additional information, such as morphology for skCSD [16]. We now want to characterize the predictive power of the combination of our setup and our selected basis, ${\tilde{b}}_{i}$. To do this we select a family of test functions, *C*^*i*^(**x**), for example Gaussian test functions, centered in different places, of multiple radii, or products of Fourier modes, etc. Then, for each *C*^*i*^ we compute $V^{i}=AC^{i}$ by forward modeling, generating a surrogate dataset. Next, we apply the standard kCSD reconstruction procedure obtaining estimation of the tested ground truth, ${\tilde{C}}^{i}$. We can then compute reconstruction error using point-wise modification of the Relative Difference Measure (RDM) proposed by [37]:

$$\begin{array}{c} {\text{err}}_{i}\left(x\right)=|\frac{{\tilde{C}}^{i}\left(x\right)}{∥{\tilde{C}}^{i}∥}-\frac{C^{i}\left(x\right)}{∥C^{i}∥}|*\frac{∥C^{i}∥}{{\text{max}}_{x\in R}\left|C^{i}\right|}, \end{array}$$ (25)

where *i* = 1, 2, … enumerates different ground truth profiles. A simple measure of reconstruction accuracy is then given by the average over these profiles:

$$\begin{array}{c} \text{Reliability}\left(x\right)≔{\left\langle {\text{err}}_{i}\left(x\right)\right\rangle }_{i\in [1,M]}=\frac{\sum_{i=1}^{M}{\text{err}}_{i}\left(x\right)}{M}. \end{array}$$ (26)

**Fig 5:**
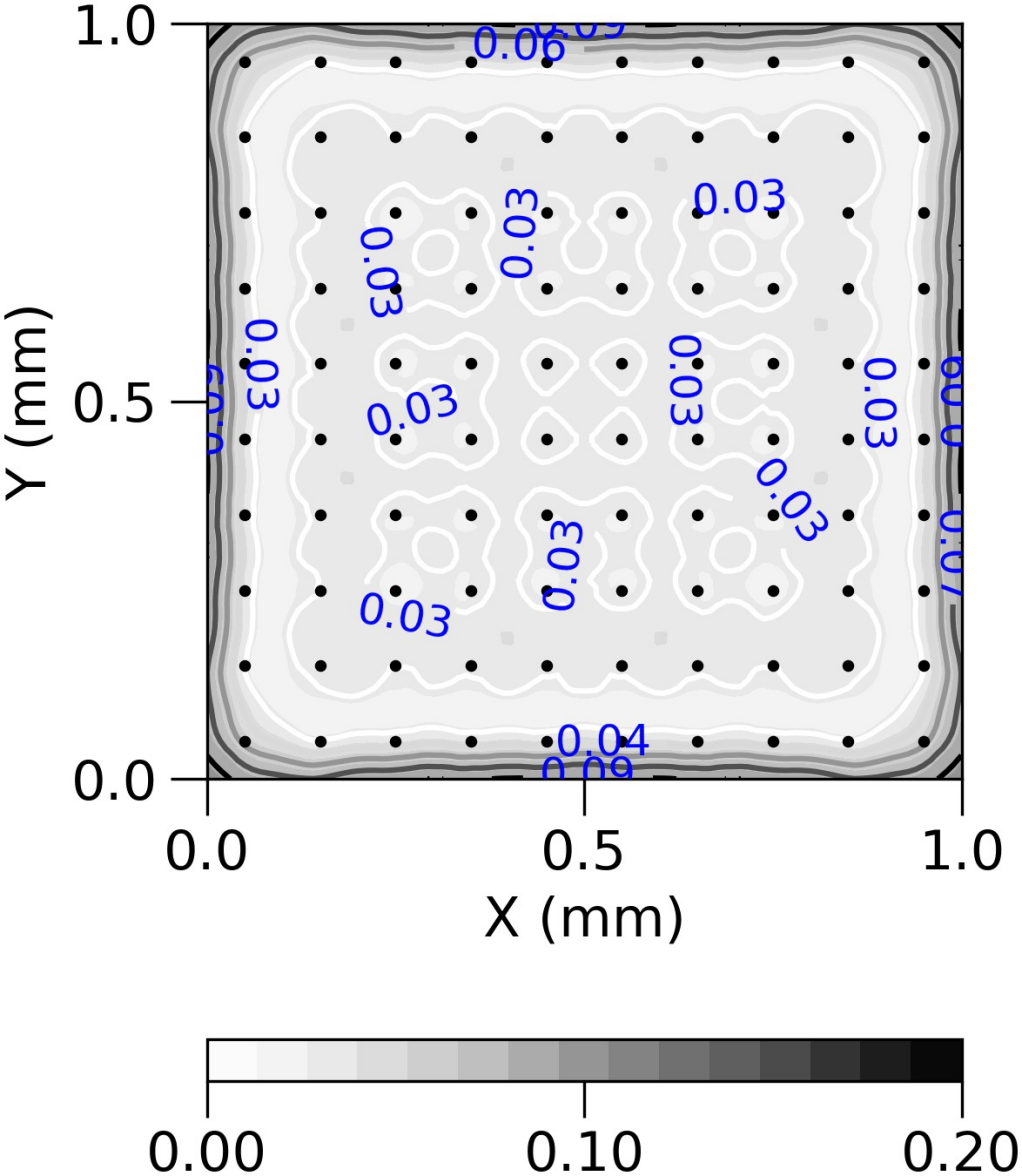
Reliability map created according to formula (25) and (26) for 10x10 regular grid of electrodes with noise-free symmetrized data. Black dots represent locations of contacts used in the study. Values on the map can be interpreted as follows: the closer to 0, the higher reconstruction accuracy might be achieved for a given measurement condition.

Fig 5 shows an example reliability map for the case of 10x10 electrode distribution.

The class of functions used were the families of small and large sources mentioned above. We used eight mirror symmetries of the grid in computation.

**Fig 6:**
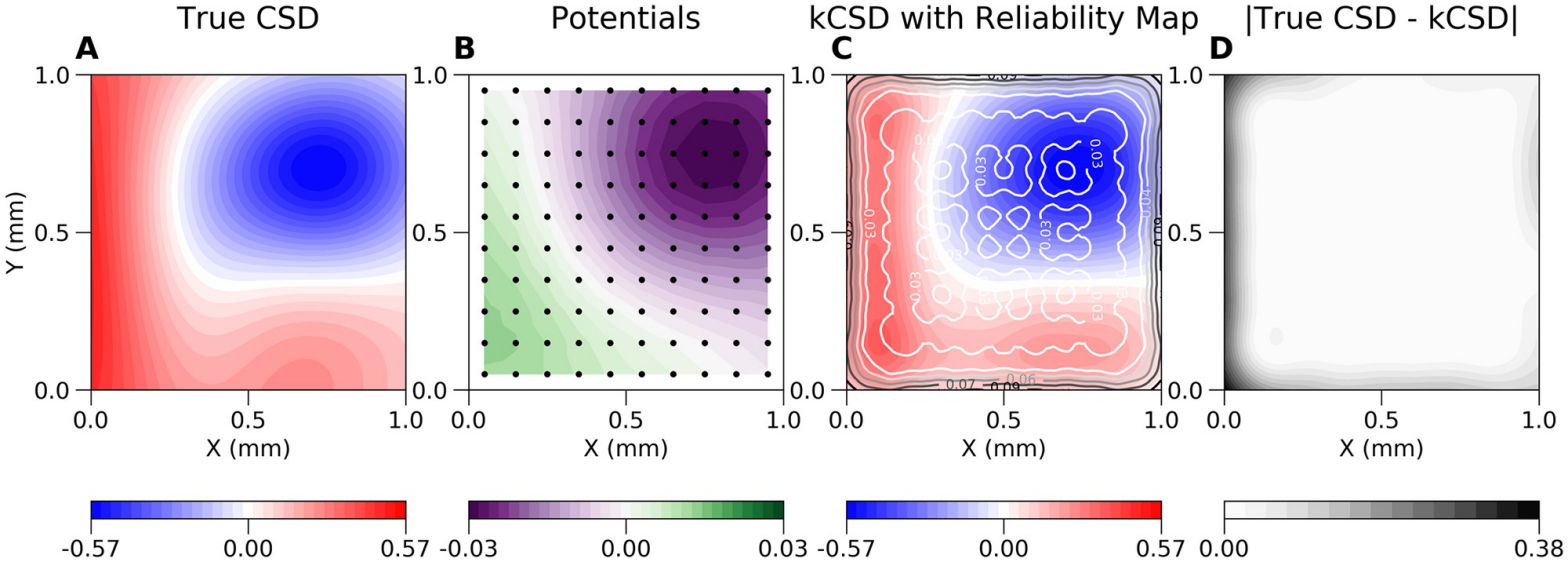
Example use of reliability maps. A) Example dipolar source (ground truth) which is used to compute the potential on a grid of electrodes shown in B). C) shows reconstructed sources superimposed on the reliability map. D) shows the difference between the ground truth and the reconstruction.

We can use the reliability map as another source of information about the precision of reconstruction, which is shown in Fig 6. In A) we show some dipolar source which is used to compute the potential on a grid of electrodes shown in B). Fig 6C) shows reconstructed sources superimposed on the reliability map. Panel D) shows the difference between the ground truth and reconstruction. Note that plots such as those shown in the panels A) and D) are feasible only for simulated or model data, where we know actual sources and use them to validate the method. On the other hand, plots shown in panel B and C represent what can be routinely computed for experimental data. This functionality is implemented in kCSD-python and illustrated in the provided tutorial.

Another interesting question is the effect of broken or missing electrodes on the reconstruction. Formally one can attempt kCSD reconstruction from a single signal but it is naive to expect much insight this way. It is thus natural to ask what information can be obtained from a given setup and what we lose when part of it becomes inaccessible.

**Fig 7:**
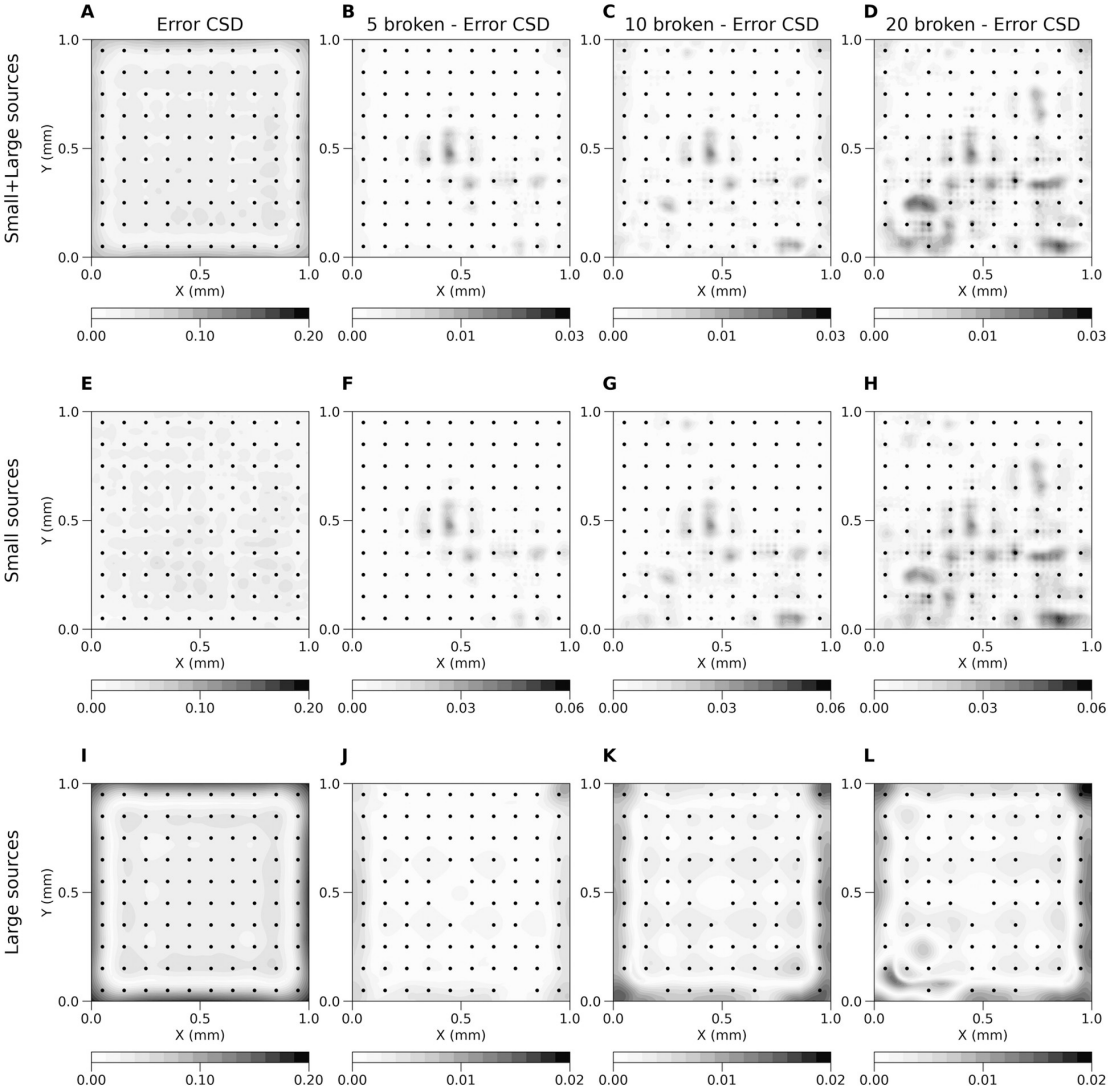
Average error (Eq 25) of kCSD method across random small and large (A), only small (E) and only large (I) sources for regular 10x10 electrodes grid and the same grid with broken 5 (B, F, J), 10 (C, G, K) and 20 (D, H, L) contacts. Plots (B, C, D, F, G, H, J, K, L) show difference between average error for regular grid and grid with broken contacts. Estimation was made in noise free scenario, *R* parameter selected in cross-validation. Black dots represent locations of contacts used in the study.

Fig 7 shows the effect of removing electrodes on the reconstruction. Fig 7A shows average error of kCSD method across many random ground truth sources for a regular grid of 10x10 electrodes. Fig 7B to D show the increase of average reconstruction error as we remove 5 (B), 10 (C) and 20 (D) contacts. To emphasize the errors we show the difference between the reliability map for the broken grid minus the original one. Note the different scales in plots B–D versus A The consecutive rows show similar results when only small sources were used (E–H), or only large sources were used (I–L). Random sources in Fig 7A are both small and large sources (mentioned in the Results). This shows, among others, as we explained, that the reliability maps depend on the test function space, however, we feel they are more intuitive to understand than the individual eigensources spanning the solution space [13].

#### The sources of error in kCSD estimation and how to deal with them

Kernel CSD method assumes a set of electrode positions and a corresponding set of recordings. Additionally, single cell kCSD requires morphology of the cell which contributed to the recordings and its position relative to the electrodes. Each of these may be subject to errors.

In analysis, we assume that the electrode positions are known precisely. This is a justified assumption in the case of multi-shaft silicon probes or integrated CMOS-MEA but not necessarily when multiple laminar probes are placed independently within the brain or for many other scenarios. For example, for SEEG electrodes in human patients used in presurgical evaluation we expect the localization errors due to the workflows used clinically to be significant. We do not provide dedicated tools to study the effects of misplaced electrodes on the reconstructed CSD, however, this can be achieved easily with the provided package if needed. The location of the cell relative to the electrodes is much more questionable, especially in 3D. Nevertheless, the necessary data to perform skCSD today are too scarce to start addressing these issues.

On the other hand we do assume that the recordings are noisy and we use regularization to counteract the effects of noise. We have no mechanism to differentiate between electrodes with varying degrees of noise to compensate this differently. However, we observed that for cases with very bad electrodes, similar results are obtained for the analysis of complete data and analysis of partial data with bad electrodes removed from the analysis. The difference was in λ selected which was larger when broken electrodes were included in the analysis. Depending on the situation, if there is a big difference in the noise visible in different channels, an optimal strategy may be to discard the noisy data and perform reconstruction from the good channels only, which kCSD permits. In the end, data analysis remains an art and a healthy dose of common sense is always recommended.

The main limitation of the method itself lies in the character of any inverse problem. Here it means that there is an infinite number of possible CSD distributions each consistent with the recorded potential. It is thus necessary to impose conditions which allow unique reconstruction and this is what every variant of CSD method is about. In kCSD this condition is the minimization of regularized prediction error. In practical terms one may think of the function space in which we are making the reconstruction. This space is spanned by the eigensources we discussed before [13]. We feel it is useful to consider both this space as well as its complement, that is the set of CSD functions whose contribution to every potential is zero. This can facilitate understanding of which features of the underlying sources can be recovered and which are inaccessible to the given setup. While for the most common regular setups, such as rectangular or hexagonal MEA grids or multi-shaft probes, intuitions from Fourier analysis largely carry over, in less regular cases this quickly becomes non-obvious.

To facilitate intuition building in the provided toolbox we include tools to compute the eigensources for a given setup. We also proposed here reliability maps, heuristic tools to build intuition regarding which parts of the reconstructed CSD can be trusted and which seem doubtful. These reliability maps are built around specific test ground truth distributions and some default parameters facilitating validation for any given setup are provided. Due to the open source nature of the provided toolbox more complex analysis is possible if the setup or experimental context require that.

## Availability and future directions

This paper introduces the kCSD-python package, an implementation of the kernel Current Source Density method [12] and its two variants ([20] and [16]). It is open source and available under the modified BSD License (3-Clause BSD) on GitHub (https://github.com/Neuroinflab/kCSD-python). It utilizes the continuous integration provided by Travis CI. It supports Python 3.8, 3.9, 3.10, and 3.11 versions and has minimal library requirements (numpy, scipy, and matplotlib). It can be installed using the Python package installer (pip) or using the Anaconda python package environment (conda). Details of the installation can be found in the package documentation at https://kcsd-python.readthedocs.io/en/latest/INSTALL.html.

The package contains a set of tools for kCSD analysis and to validate the results obtained from this analysis. To facilitate the uptake of this resource, the package comes with extensive tutorials implemented in Jupyter Notebooks. These tutorials allow users to test different configurations of current sources and electrodes to see the method in action. The users can analyze their data or explore the method with data generated *in silico*. These provisions illustrate the advantages and limitations of kCSD method to its users. The tutorials can also be accessed without any installation on a web browser via Google Colaboratory [Google CoLab (RRID:SCR_018009)]. The package is extensively documented (https://kcsd-python.readthedocs.io) and includes all the necessary scripts to generate the figures in this manuscript.

An extensive tutorial overview of the kCSD-python package is provided in S1 Text. Its goal is to show how to use kCSD-python to perform CSD analysis, how to apply the provided analysis tools, and how to validate the results. We first consider a regular grid of ideal (noise-free) electrodes, where we compute the potentials from a known test source (the ground truth). We then use these potentials to reconstruct the sources which we compare with ground truth (Basic features). Then, we explore the effects of noise on the reconstruction and test the robustness of the method (Noisy electrodes). In the final part of the tutorial we look at how the errors in the estimation depend on the sources and the electrode configuration by testing the effects of broken electrodes on reconstruction (Broken electrodes).

S1 Fig shows error propagation maps, which we introduce below, for a 1D regular grid of 12 electrodes. S2 Fig shows an example of 3D kCSD source reconstruction. S3 Fig shows an example of skCSD reconstruction (single cell kernel CSD) which corresponds to Fig 8 from [16]. The simulation, reconstruction, and visualization, have all been re-implemented in Python.

We also provide some pre-computed examples of CSD estimations using our library for users to explore. We provide these as .pdf files available at https://doi.org/10.18150/KRYNCA. Here, the files small_srcs_3D_all.pdf and large_srcs_3D_all.pdf show 100 example setups of 3D kCSD reconstructions from small and large sources. Similarly, files small_srcs_all.pdf and large_srcs_all.pdf show 100 example setups of 2D kCSD reconstructions from small and large sources. “Smallness” and “largeness” of sources are defined by the ratios of typical spatial scales of the source relative to interelectrode distances.

Throughout this work and in the toolbox we assumed the purely ohmic character of the tissue. This has been debated in recent years [8, 38, 39] and it is true that more complex biophysical models of the tissue, taking into account frequency-dependent conductivity or diffusion currents, could influence the practice of source reconstruction or its interpretation. However, the available data indicate that in the range of frequencies of physiological interest these effects are small. While one should keep eyes open on the new data as they become available and keep in mind the different possible sources which may affect the reconstruction or interpretation, we believe that the traditional view of ohmic tissue is an adequate basis for typical experimental situations and going beyond that would probably require additional dedicated measurement for the experiment at hand which may not always be feasible. For example, as we discussed in [20], the specimen variability of the cortical conductivity in the rat is much bigger than the variability between different directions within a given rat [40]. This means that unless we have conductivity measurements for our specific rat we are probably making smaller errors assuming isotropic conductivity than taking different values from the literature. We feel there is not enough data to justify the inclusion of more complex terms in the standard CSD analysis to be applied throughout the brains and species.

In this manuscript and in the kCSD-python toolbox we also assumed constant conductivity (with the exception of the MoI case). We are convinced this is a reasonable approximation for typical depth recordings. In general, however, this approximation needs to be justified or alternative models of tissue need to be considered. In principle, the kCSD method can be applied to a variety of tissue models as long as the basis potentials can be computed from the basis sources while incorporating the geometric and conductivity changes.

For example, [20] considered a cortical slice placed on a microelectrode array (MEA) in which they included the geometry of the slice and modeled the saline-slice interface with changing conductivity in the forward model. They found that the Method of Images (MoI) gives a good approximation to the full solution obtained using a finite-element model (FEM). This approximation was incorporated within the kCSD method as the MoIkCSD variant and is available in the kCSD-python package.

It is possible to generalize kCSD to reconstruct sources from recordings of multiple electrical modalities—LFP, ECoG, and EEG. In this case one needs to include the head geometry and the changing tissue properties within the forward model and in the kCSD method. The anisotropic (white matter tracts) and inhomogeneous (varying between skull, cerebrospinal fluid, gray matter, and white matter) electrical conductivity changes can be approximated using data obtained with imaging techniques such as MRI, CT or DTI. Such sophisticated head models require numerical solutions such as finite element modeling (FEM) to compute the basis potentials from the basis sources. We are currently working on this approach to make it generic for any animal head and to eventually utilize it as a source localization method for human data, for example, to localize foci of pharmacologically intractable epilepsy seizures in humans. We call this extension kernel Electrical Source Imaging (kESI).

## Supporting information

S1 Text kCSD-python package tutorial. (PDF)

S1 Fig Error propagation maps for 1D regular grid of 12 electrodes. Every panel represents the CSD contribution (red line) of the potential measured at the corresponding electrode, for which the potential is 1 (green line).
(TIF)

S2 Fig An example of 3D kCSD source reconstruction. Each column shows five consecutive parallel cuts through a box of size 1. A) Ground truth for the CSD seed of 16. B) Estimated potential; black dots indicate electrodes where potential is collected for further reconstruction. C) 3D kCSD reconstruction from the measured potentials, λ = 0. D) 3D kCSD reconstruction with cross-validation.
(TIF)

S3 Fig An example of skCSD reconstruction [16] of somatic current injection together with random synaptic input patterns for a retinal ganglion cell model. A) Somatic membrane potential. B) Current density and its C) skCSD reconstruction in the segment space. Projection of D) ground truth and E) skCSD reconstruction on the neuron’s morphology at 5 s of the simulation. We simulated a multicompartmental model of a mouse retinal ganglion cell (morphology [41] obtained from NeuroMorpho.Org [42]) with Hodgkin-Huxley sodium, potassium, and leakage channels in the soma (hh mechanism) in NEURON simulation environment. For calculation of the measured extracellular potentials we used LFPy package [43]. The model neuron was stimulated by an injection of oscillatory current to the soma (with frequency of 24.5 1/ms and amplitude of 3.6 nA) together with random synaptic inputs (weight of 0.04 *μS*) to the dendritic tree. The activity of the model neuron was measured by a rectangular grid of 100 electrodes (10 × 10, -400 *μm* × 400*μm*). The figure corresponds to Fig 8 from [16].
(TIF)
