# Supplementary material for: kCSD-python, reliable current source density estimation with quality control
Source: PLoS Comput Biol. 2024 Mar 14;20(3):e1011941. doi: 10.1371/journal.pcbi.1011941 (PMC10965101; doi:10.1371/journal.pcbi.1011941)
Supplement: S1 Text — (PDF) [file pcbi.1011941.s001.pdf]

kCSD-python, reliable current source density estimation with quality control

Chaitanya Chintaluri[1,2,¶], Marta Bejtka[1,¶], Władysław Średniawa[1,3], Michał Czerwiński[1], Jakub M. Dzik[1], Joanna Jędrzejewska-Szmek[1], Daniel K. Wójcik[1,*]

1 Laboratory of Neuroinformatics, Nencki Institute of Experimental Biology of Polish Academy of Sciences, Warsaw, Poland
2 Institute of Science and Technology Austria, Klosterneuburg, Austria
3 University of Warsaw, Faculty of Biology, Department of Zoology, Warsaw, Poland

[*] d.wojcik@nencki.edu.pl
[¶] joint first authors

Supporting information

S1 Text: kCSD-python package tutorial

In this section we first illustrate the use of the kCSD package for CSD reconstruction in the simplest case of a regular 2D square grid. This is a simplified version of a slice on a microelectrode array [1], or a planar silicone probe within the brain, where we assume constant conductivity in the whole space. In the following sections we show how we validate our methods and what kind of diagnostics we find useful in the analysis of experimental data. This tutorial is available as a Jupyter Notebook and can also be accessed through a web browser without installation. For more details, see 'Availability and future directions'.

Basic features

We start with the basic CSD estimation on a regular grid. First, we define a region of interest. Then, using predefined test functions for the current sources, we place a ground truth current source in this region. We define the distribution of electrodes. Assuming ideal electrodes, we compute the potential generated by the selected current sources as measured at the electrodes. Given these potentials and the electrode locations we estimate the current source density using kCSD. As a final step, we perform cross-validation to avoid overfitting. Since we know the ground truth used to generate the potentials that were used in the kCSD estimation, we can compare the ground truth to the estimate and see the reconstruction accuracy.

Defining region of interest

```
In [1]: %matplotlib inline
        import numpy as np
        csd_at = np.mgrid[0.:1.:101j,
                          0.:1.:101j]
        csd_x, csd_y = csd_at
```

We define the region of interest between `0` and `1` in the `xy` plane with a resolution of `101` points in each dimension. We will assume the distance is given in mm, so we want to perform a reconstruction on a square patch of $1 mm^2$ size.

Setting up the ground truth The `kCSD-python` library provides functions to generate test sources which can be imported from the `csd_profile` module. Here we use the `gauss_2d_small` function to generate two-dimensional Gaussian sources which are small in the scale set by the interelectrode distance. The other implemented option for two-dimensional test sources is the `gauss_2d_large` function. To generate the exact same sources in each run we must invoke this function using the same random seed which is stored in the `seed` variable. For simplicity, these current sources are static and do not change with time. We visualize the current sources as a heatmap.

```
In [2]: from kcsd import csd_profile as CSD
        CSD_PROFILE = CSD.gauss_2d_small
        true_csd = CSD_PROFILE(csd_at, seed=15)
```

The code below displays this test source as the True CSD. For convenience we define this as a function `make_plot`. The output for this code is shown in Fig 1A.

```
In [3]: import matplotlib.pyplot as plt
        import matplotlib.cm as cm

        def make_plot(xx, yy, zz, title='True CSD', cmap=cm.bwr):
            fig = plt.figure(figsize=(7, 7))
            ax = plt.subplot(111)
            ax.set_aspect('equal')
            t_max = np.max(np.abs(zz))
            levels = np.linspace(-1 * t_max, t_max, 32)
            im = ax.contourf(xx, yy, zz, levels=levels, cmap=cmap)
            ax.set_xlabel('X (mm)')
            ax.set_ylabel('Y (mm)')
            ax.set_title(title)
            ticks = np.linspace(-1 * t_max, t_max, 3, endpoint=True)
            plt.colorbar(im, orientation='horizontal', format='%.2f',
                         ticks=ticks)
            return ax
        make_plot(csd_x, csd_y, true_csd, title='True CSD', cmap=cm.bwr)
```

Place electrodes We now define the virtual electrodes within the region of interest. We place them between `0.05` mm and `0.95` mm of the region of interest, with a resolution of `10` (as indicated by `10j` in `mgrid`) in each dimension, totalling `100` electrodes. Notice that the electrodes do not span the entire region of interest. Although in this example the electrodes are distributed on a regular grid, this is not required by the kCSD method as it can handle arbitrary distributions of electrodes.

```
In [4]: ele_x, ele_y = np.mgrid[0.05: 0.95: 10j,
                                0.05: 0.95: 10j]
        ele_pos = np.vstack((ele_x.flatten(), ele_y.flatten())).T
```

Compute potential To obtain the potential, `pots`, at the given electrode positions due to the current sources that were placed in the previous steps we use the function `forward_method`. We assume the sources are localized within a slab of tissue of thickness 2`h` on top the MEA (See [1, 2] and Methods). We also assume an infinite homogeneous medium of conductivity `sigma` equal to `1` S/m. Finally, we assume that the electrodes are ideal, point-size, and noise-free.

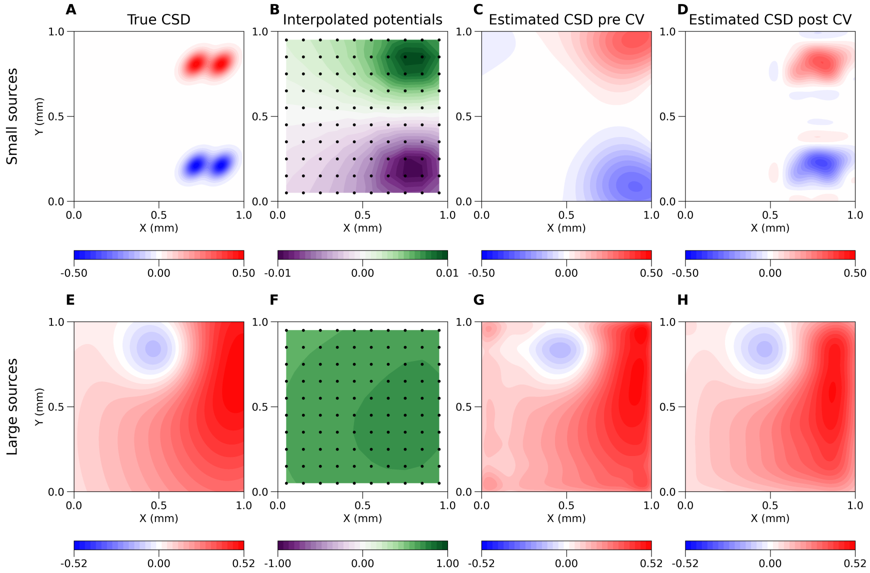

Fig 1. Basic features tutorial A) Shows the ground truth (True CSD), here, two-dimensional small Gaussian current sources, for the CSD seed of 15. B) The interpolated potentials generated by this current source are shown, the electrodes are displayed as black dots. C) CSD estimated with kCSD using the potentials from the electrode positions, without cross-validation. D) Same as C but cross-validation was used. E-H) Analogous to A–D, except large Gaussian current sources for seed 6 were used.

```
In [5]: from scipy.integrate import simps

        def integrate_2d(csd_at, true_csd, ele_pos, h, csd_lims):
            csd_x, csd_y = csd_at
            xlin = csd_lims[0]
            ylin = csd_lims[1]
            Ny = ylin.shape[0]
            m = np.sqrt((ele_pos[0] - csd_x)**2 + (ele_pos[1] - csd_y)**2)
            m[m < 0.0000001] = 0.0000001
            y = np.arcsinh(2 * h / m) * true_csd
            integral_1D = np.zeros(Ny)
            for i in range(Ny):
                integral_1D[i] = simps(y[:, i], ylin)
            return simps(integral_1D, xlin)

        def forward_method(ele_pos, csd_at, true_csd):
            pots = np.zeros(ele_pos.shape[0])
            xlin = csd_at[0, :, 0]
            ylin = csd_at[1, 0, :]
            h = 50.  # distance between the electrode plane and midslice
            sigma = 1.0 # S/m
            for ii in range(ele_pos.shape[0]):
                pots[ii] = integrate_2d(csd_at, true_csd,
                                        [ele_pos[ii][0], ele_pos[ii][1]], h,
                                        [xlin, ylin])
```

```
        return pots / 2 * np.pi * sigma

    pots = forward_method(ele_pos, csd_at, true_csd)
```

To visualize the potential, we interpolate a hundred values computed at the electrodes positions with the `interpolate.griddata` function. Note that the kCSD estimation uses only the potential recorded at the positions of the electrodes. To distinguish between the potentials and CSD plots we use different colormaps. The electrodes are marked with dots in this plot. The output from this step is shown in Fig 1B.

```
In [6]: from scipy.interpolate import griddata

        def grid(x, y, z):
            x = x.flatten()
            y = y.flatten()
            z = z.flatten()
            xi, yi = np.mgrid[min(x):max(x):100j,
                              min(y):max(y):100j]
            zi = griddata((x, y), z, (xi, yi), method='linear')
            return xi, yi, zi

        pot_X, pot_Y, pot_Z = grid(ele_pos[:, 0], ele_pos[:, 1], pots)
        ax = make_plot(pot_X, pot_Y, pot_Z, title='Interpolated potentials',
                       cmap=cm.PRGn)
        ax.scatter(ele_pos[:, 0], ele_pos[:, 1], 10, c='k')
```

kCSD method Here we illustrate the most basic estimation of CSD with the `kcsd` library. Since our example is two dimensional the relevant method is `KCSD2D`. For convenience, we encapsulate the actual method call with parameters being set inside a function `do_kcsd`. We first set the `h` and `sigma` parameters of the forward model. Then we restrict the potentials to the first time point of the recording. For typical experimental data the shape of this matrix would be $N_{ele} \times N_{time}$, where N_{ele} is the number of electrodes and N_{time} is the total number of recorded time points. Next, we call the `KCSD2D` class with the relevant parameters. The only required parameters are the electrode positions, `ele_pos`, and the potentials they see, `pots`. We can also provide here the parameters for the forward model, `h` and `sigma`. We define a rectangular region of estimation by setting the values `xmin`, `xmax` and `ymin`, `ymax`. The number of basis functions, `n_src_init` is set to `1000`, basis functions are of the type `gauss`, and the width of the Gaussian basis source `R_init` is set to be `1`. Finally, the estimated CSD is stored as `est_csd`.

```
In [7]: from kcsd import KCSD2D

        def do_kcsd(ele_pos, pots):
            h = 50.  # slice thickness
            sigma = 1.0 # S/m
            pots = pots.reshape((-1, 1)) # first time point
            return KCSD2D(ele_pos, pots, h=h, sigma=sigma,
                          xmin=0.0, xmax=1.0,
                          ymin=0.0, ymax=1.0,
                          n_src_init=1000, src_type='gauss', R_init=1.)

        k = do_kcsd(ele_pos, pots)
        est_csd = k.values('CSD')
```

Estimated current sources are shown in Fig 1C. Compare this to the True CSD obtained before, Fig 1A. Observe that the estimation is not very faithful. This is caused by the ground truth varying significantly in the scale of a single inter-electrode distance. In the next step we will use cross-validation to select better reconstruction parameters.

```
In [8]: make_plot(k.estm_x, k.estm_y, est_csd[:, :, 0],  # First time point
                  title='Estimated CSD without CV', cmap=cm.bwr)
```

Cross-validation Leave-one-out cross-validation is performed with a single-line command. In this procedure we scan a range of `R` values which set the size of the Gaussian basis functions and the regularization parameter λ values. At the end of this step we obtain the optimal parameters that would correct for overfitting. The function outputs the progress of the cross-validation step and displays the optimal candidates in the last line. Alternatively, one could use the L-curve method to find these optimal parameters. Fig 1D shows the kCSD reconstruction obtained after cross-validation. We find that this estimation of the current sources resembles the True CSD better.

```
In [9]: k.cross_validate(Rs=np.linspace(0.01, 0.15, 15))
        est_csd = k.values('CSD')
```

```
No lambda given, using defaults
Cross validating R (all lambda) : 0.01
Cross validating R (all lambda) : 0.02
...
Cross validating R (all lambda) : 0.15
R, lambda : 0.11 1.46779926762e-06
```

```
In [10]: make_plot(k.estm_x, k.estm_y, est_csd[:, :, 0],  # First time point
                   title='Estimated CSD with CV', cmap=cm.bwr)
```

Noisy electrodes

Until now we assumed noise-free data, however, experimental data are always noisy. In this section we investigate how noise affects the kCSD estimation. We first show how to compute the reliability map which we introduced before, Eq (26). Then we discuss reproducible generation of noisy data with varying noise amplitude. Finally, we study the error in the reconstruction as a function of changing noise levels.

Reconstruction quality measure To assess the estimation quality we measure the point-wise difference between the true sources and the sources reconstructed with the kcsd. We define a function `point_errors` which takes the `true_csd` and the `estimated_csd` as the inputs, normalizes them individually, and computes the Frobenius norm of their difference.

```
In [11]: def point_errors(true_csd, est_csd):
             true_csd_r = true_csd.reshape(true_csd.size, 1)
             est_csd_r = est_csd.reshape(est_csd.size, 1)
             epsilon = np.linalg.norm(true_csd_r)/np.max(abs(true_csd_r))
             err_r = abs(est_csd_r/(np.linalg.norm(est_csd_r)) -
                         true_csd_r/(np.linalg.norm(true_csd_r)))
             err_r *= epsilon
             return err_r.reshape(true_csd.shape)

         error_ideal = point_errors(true_csd, est_csd)
```

We visualize this difference as before, except we use a greyscale colormap to display the intensity of the reconstruction error. For convenience, we define the plotting in a function called `make_error_plot`. The output from this step is shown in Fig 2A.

```
In [12]: def make_error_plot(xx, yy, error, title='Error CSD'):
             fig = plt.figure(figsize=(7, 7))
             ax = plt.subplot(111)
             ax.set_aspect('equal')
             t_max = np.max(np.abs(error))
             levels = np.linspace(0, t_max, 32)
             im = ax.contourf(xx, yy, error, levels=levels, cmap=cm.Greys)
             ax.set_xlabel('X (mm)')
             ax.set_ylabel('Y (mm)')
             ax.set_title(title)
             ticks = np.linspace(0, t_max, 3, endpoint=True)
             plt.colorbar(im, orientation='horizontal', format='%.2f',
                          ticks=ticks)
             return ax

         make_error_plot(k.estm_x, k.estm_y, error_ideal,
                         title='Error CSD, no noise')
```

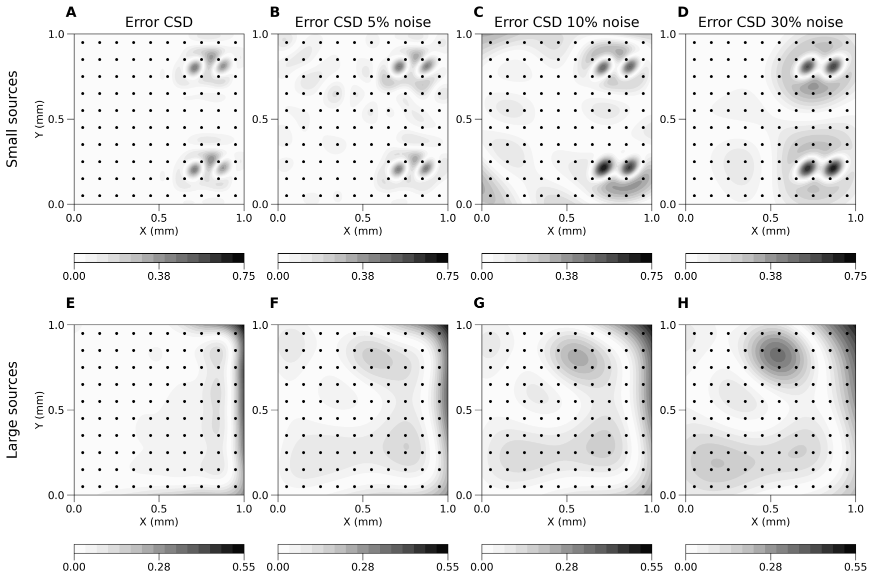

Fig 2. Noisy electrodes. A) The error between the True CSD and the estimation obtained with kCSD for a 2 dimensional small Gaussian current source, using the csd seed of 15. The electrodes in this case are assumed to be noise-free. B, C, D) Same as A, however, noise is added to the recorded potentials, whose magnitude is 5%, 10%, or 30%, respectively. E-H) Analogous to A–D, except in this case large Gaussian sources with seed 6 were used.

Noise definition To study resilience of the reconstruction against the noise in a controlled way we seed the random number generator in the function `add_noise`. We consider normally distributed noise with the mean and standard deviation set by reference to the recorded potentials.

```
In [13]: def add_noise(pots, noise_level=0, noise_seed=23):
             rstate = np.random.RandomState(noise_seed)
             noise = noise_level*0.01*rstate.normal(np.mean(pots),
                                                    np.std(pots),
                                                    size=(len(pots), 1))
             return pots + noise.reshape(pots.shape)

         pots_noise = add_noise(pots, noise_level=15, noise_seed=23)
```

Source reconstruction from noisy data With these tools we can study the effects of noise on the reconstruction. We now generate noise for a given noise level between 0 and 100, add it to the simulated potential, and estimate CSD from these noisy potentials. We can then use the error plots to compare the reconstruction with the True CSD. Notice that the parameters giving best reconstruction obtained for noisy data in general will be different from those obtained for clean potentials to compensate for noise.

```
In [14]: k_noise = do_kcsd(ele_pos, pots_noise)
         k_noise.cross_validate(Rs=np.linspace(0.01, 0.15, 15))
         estm_csd_noise = k_noise.values('CSD')
         error_noise = point_errors(true_csd, estm_csd_noise)
```

```
No lambda given, using defaults
Cross validating R (all lambda) : 0.01
Cross validating R (all lambda) : 0.02
...
Cross validating R (all lambda) : 0.15
R, lambda : 0.01 0.00110069417125
```

We can display this error with the `make_error_plot` plotting function which we defined earlier. Changing the `noise_level` and the `noise_seed` affects the reconstruction, but the error depends also on the sources, so changing the True CSD type to a `gauss_2d_large` or changing `csd_seed` will lead to different results. This is illustrated in Fig 2A–D for small Gaussian sources, and Fig 2E–H for large Gaussian sources, with varying noise levels. The actual ground truth and reconstructions are shown in Fig 1.

```
In [15]: make_error_plot(k_noise.estm_x, k_noise.estm_y, error_noise,
                         title='Error CSD, with noise')
```

Broken electrodes

It is often the case that due to experimental constraints, some subset of recordings are discarded in the final analysis. This can happen when some electrodes are used for stimulation and cannot be used for recording, or due to bandwidth limitations requiring a compromise between sampling rates and the number of simultaneously recording electrodes, or in the event of an electrode break down. In this section of the tutorial we discuss how to handle such cases and to estimate the errors in reconstruction despite the loss of recordings. We first show how we remove recordings from selected (broken) electrodes from considered data. Then we calculate the estimation error for a given source for data from such a damaged setup. Finally, we compute the average error across many sources from such an incomplete setup. Note that kCSD reconstruction is designed to work with arbitrary electrode setups and removing specific electrodes does not change the situation significantly. We focus on broken electrodes as it is a common situation in practice and deserves consideration. This may be used to gain intuition

regarding ways in which CSD reconstruction may go wrong, due to slight disturbances in a familiar setup.

Remove broken electrodes To test the effects of removed electrodes on reconstruction from a given setup we simulate this with a function `remove_electrodes` that takes all the electrode positions for this setup and the number of electrodes that are to be removed. In this example we remove the electrodes randomly. As we did previously, to facilitate repeatability we also pass a `broken_seed` variable, so that at each subsequent run the same electrodes are discarded. By changing this seed we select a different set of electrodes for removal.

```
In [16]: def remove_electrodes(ele_pos, num_broken, broken_seed=42):
             rstate = np.random.RandomState(broken_seed)
             rmv = rstate.choice(ele_pos.shape[0], num_broken, replace=False)
             ele_pos = np.delete(ele_pos, rmv, 0)
             return ele_pos
         # Discard 5 electrodes of 100
         ele_pos_new = remove_electrodes(ele_pos, 5)
```

Error in estimation with broken electrodes After removing the broken electrodes we compute the estimation error to gauge the effect of electrode removal on reconstruction. Here, a fuction `calculate_error` takes a `csd_seed` as an input, which selects a specific ground truth source, and all the remaining electrode positions, `ele_pos`. The function computes the True CSD for a `gauss_2d_small` type source, computes the potential at these electrode locations, performs kcsd estimation from these data, and computes the error in the estimation of the true csd.

```
In [17]: def calculate_error(csd_seed, ele_pos):
             true_csd = CSD_PROFILE(csd_at, seed=csd_seed)
             pots = forward_method(ele_pos, csd_at, true_csd)
             k = do_kcsd(ele_pos, pots)
             k.cross_validate(Rs=np.linspace(0.01, 0.15, 15))
             err = point_errors(true_csd, k.values('CSD'))
             return k, err

         k, err = calculate_error(csd_seed=15, ele_pos=ele_pos_new)
```

```
No lambda given, using defaults
Cross validating R (all lambda) : 0.01
Cross validating R (all lambda) : 0.02
...
Cross validating R (all lambda) : 0.15
R, lambda : 0.14 1.33352143216e-05
```

Below (Fig 3) we plot these errors. We also display the electrodes which were used in the kcsd estimation.

```
In [18]: ax = make_error_plot(k.estm_x, k.estm_y, err,
                              title='Error CSD, 5 broken electrodes')
         ax.scatter(ele_pos_new[:, 0], ele_pos_new[:, 1], 10, c='k')
```

Average error for multiple sources As we can see, the estimation error depends on the test current sources used. To better understand the effects of the setup we compute the average error across multiple sources. As an example here we show this for

two seeds. In principle, any type and number of sources may be tested, as we showed before in the analysis of reliability maps. This step is computationally expensive, however, it would normally be carried out only once for a given electrode design configuration. We believe this approach offers useful diagnostics and builds intuition regarding the estimation power for the given setup.

```
In [19]: seed_list = range(15, 17)
         error_list = []
         for csd_seed in seed_list:
             k, err = calculate_error(csd_seed=csd_seed, ele_pos=ele_pos_new)
             error_list.append(err)
         avg_error = sum(error_list) / len(error_list)
```

```
No lambda given, using defaults
Cross validating R (all lambda) : 0.01
...
Cross validating R (all lambda) : 0.15
R, lambda : 0.14 1.33352143216e-05
No lambda given, using defaults
Cross validating R (all lambda) : 0.01
...
Cross validating R (all lambda) : 0.15
R, lambda : 0.04 3.48070058843e-15
```

In Fig 3A–D we show this for the case of 0, 5, 10, and 20 broken electrodes, when the average error for 100 small Gaussian sources was considered. In Fig 3E–H we show the same for large Gaussian sources.

```
In [23]: ax = make_error_plot(k.estm_x, k.estm_y, avg_error,
                              title='Average Error, 5 broken electrodes')
         ax.scatter(ele_pos_new[:, 0], ele_pos_new[:, 1], 10, c='k')
```

References

1. Ness TV, Chintaluri C, Potworowski J, Łęski S, Głąbska H, Wójcik DK, et al. Modelling and analysis of electrical potentials recorded in microelectrode arrays (MEAs). Neuroinformatics. 2015;13(4):403–426.

2. Łęski S, Pettersen KH, Tunstall B, Einevoll GT, Gigg J, Wójcik DK. Inverse Current Source Density method in two dimensions: Inferring neural activation from multielectrode recordings. Neuroinformatics. 2011;9(4):401–425. doi:10.1007/s12021-011-9111-4.

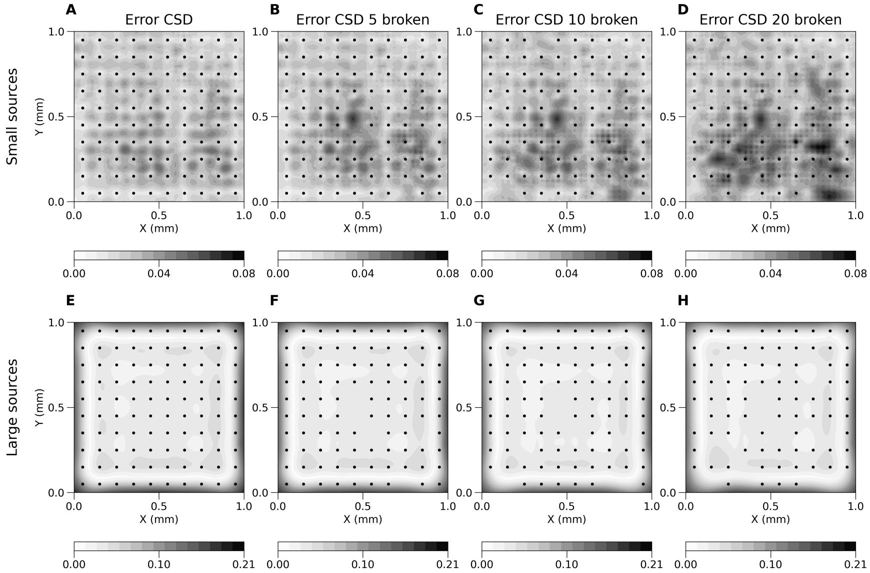

Fig 3. Broken electrodes. A) Shows the average error between the True CSD and the CSD estimated with kcsd for 100 random small Gaussian current sources. B) The same average error as in A, except in this case 5 electrodes were discarded in the estimation. Likewise for C and D, where 10 and 20 electrodes out of the 100 were considered broken. E-H) analogous to A–D, except in this case we show the averages for 100 large Gaussian current sources.
