# Supplementary figures and images for: kCSD-python, reliable current source density estimation with quality control
Source: PLoS Comput Biol. 2024 Mar 14;20(3):e1011941. doi: 10.1371/journal.pcbi.1011941 (PMC10965101; doi:10.1371/journal.pcbi.1011941)

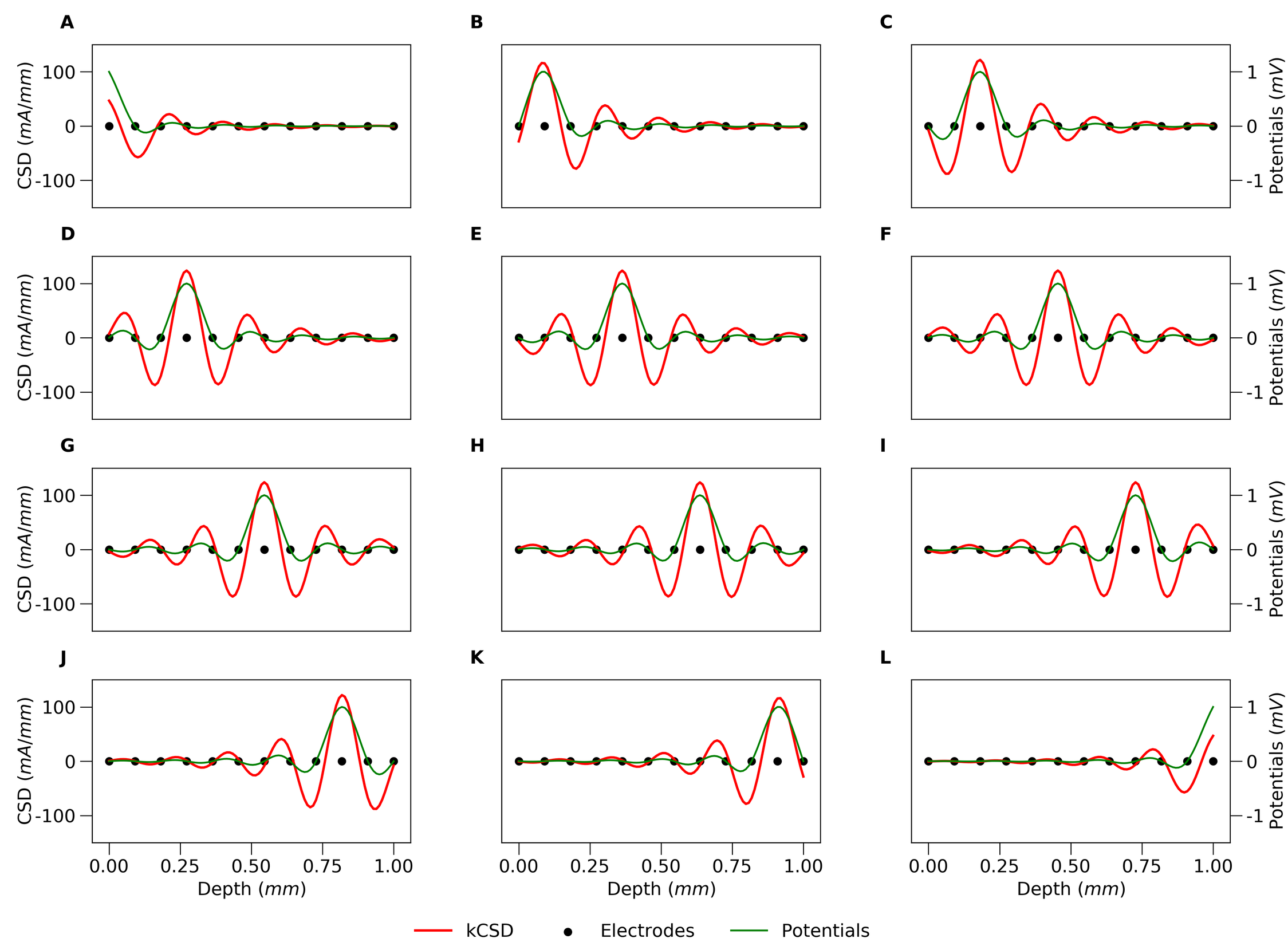

Supplement: S1 Fig — Every panel represents the CSD contribution (red line) of the potential measured at the corresponding electrode, for which the potential is 1 (green line). (TIF) [file pcbi.1011941.s002.tif]

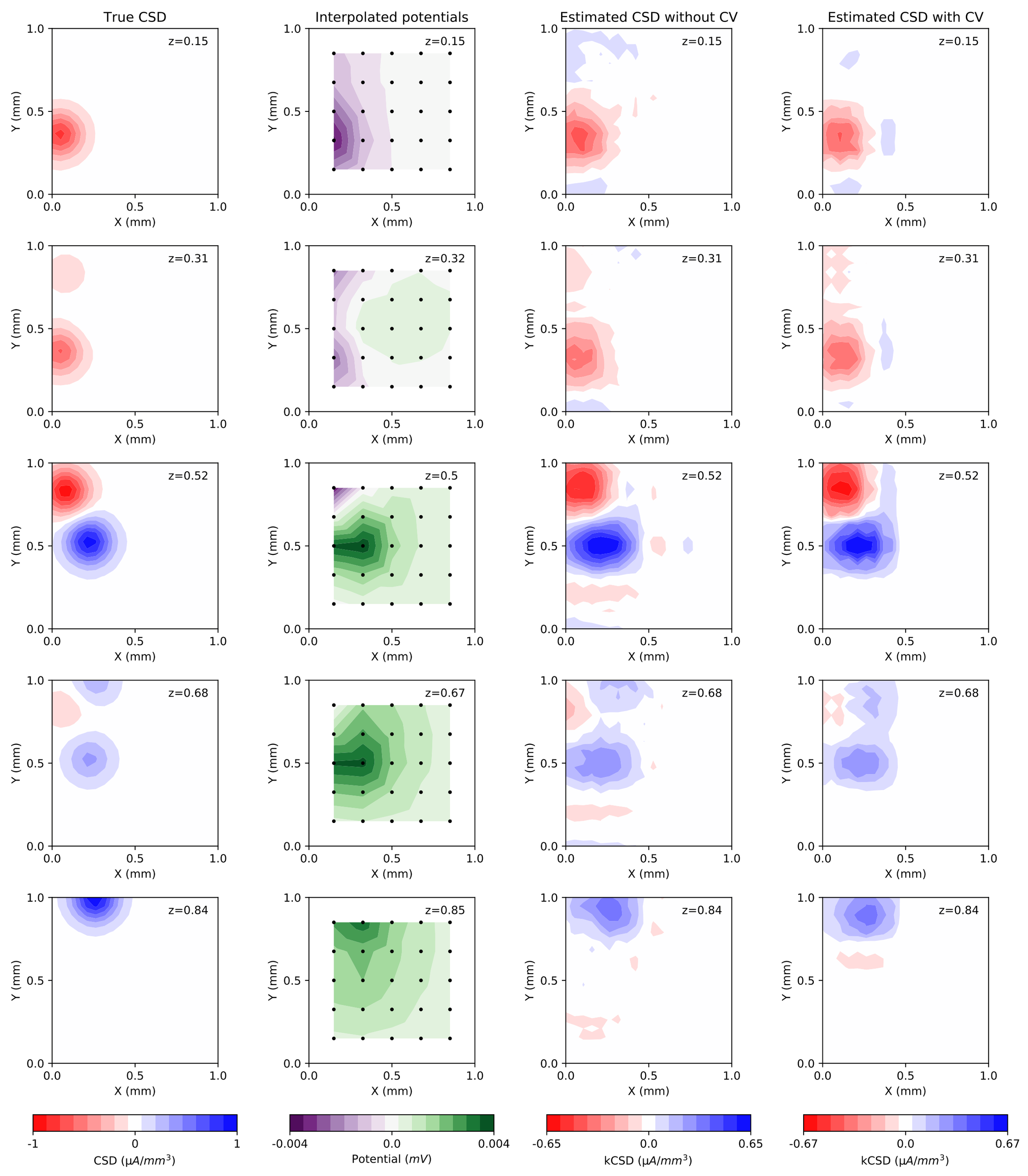

Supplement: S2 Fig — Each column shows five consecutive parallel cuts through a box of size 1. A) Ground truth for the CSD seed of 16. B) Estimated potential; black dots indicate electrodes where potential is collected for further reconstruction. C) 3D kCSD reconstruction from the measured potentials, λ = 0. D) 3D kCSD reconstruction with cross-validation. (TIF) [file pcbi.1011941.s003.tif]

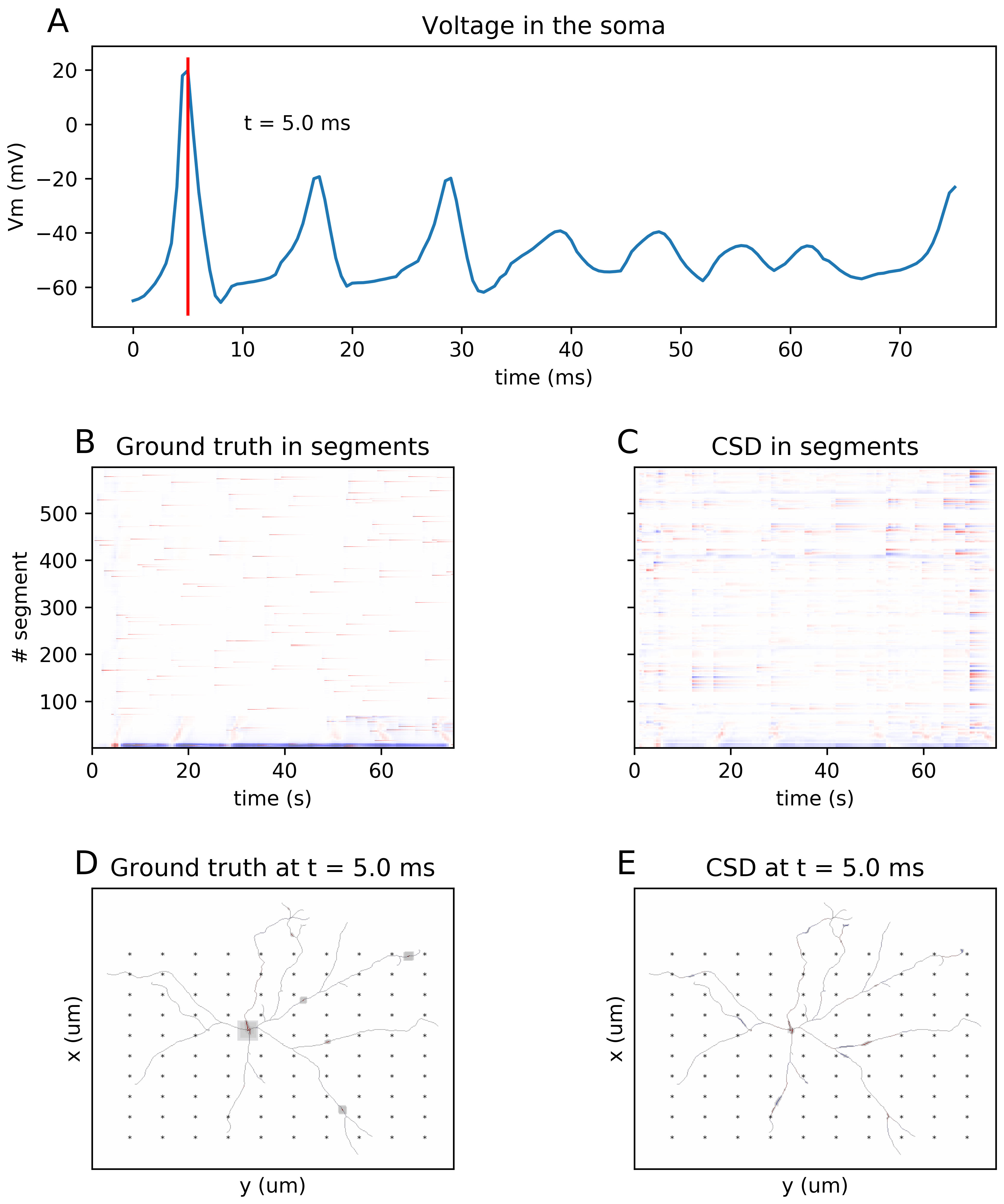

Supplement: S3 Fig — A) Somatic membrane potential. B) Current density and its C) skCSD reconstruction in the segment space. Projection of D) ground truth and E) skCSD reconstruction on the neuron’s morphology at 5 s of the simulation. We simulated a multicompartmental model of a mouse retinal ganglion cell (morphology [41] obtained from NeuroMorpho.Org [42]) with Hodgkin-Huxley sodium, potassium, and leakage channels in the soma (hh mechanism) in NEURON simulation environment. For calculation of the measured extracellular potentials we used LFPy package [43]. The model neuron was stimulated by an injection of oscillatory current to the soma (with frequency of 24.5 1/ms and amplitude of 3.6 nA) together with random synaptic inputs (weight of 0.04 μS) to the dendritic tree. The activity of the model neuron was measured by a rectangular grid of 100 electrodes (10 × 10, -400 μm × 400μm). The figure corresponds to Fig 8 from [16]. (TIF) [file pcbi.1011941.s004.tif]
